# Volatility, correlation and risk spillover effect between freight rates in BCI and BPI markets: Evidence from static and dynamic GARCH-Copula and dynamic CoVaR models

**DOI:** 10.1371/journal.pone.0315167

**Published:** 2025-01-16

**Authors:** Yuye ZOU, Jing XU, Yanhui CHEN

**Affiliations:** College of Economics and Management, Shanghai Maritime University, Shanghai, China; Vellore Institute of Technology, INDIA

## Abstract

The dry bulk shipping market plays a crucial role in global trade. To examine the volatility, correlation, and risk spillover between freight rates in the BCI and BPI markets, this paper employs the GARCH-Copula-CoVaR model. We analyze the dynamic behavior of the secondary market freight index for dry bulk cargo, highlighting its performance in a complex financial environment and offering empirical support for the shipping industry and financial markets. The findings reveal that: (1) There are significant differences in correlation across various routes, with the correlation between BCI and BPI routes fluctuating over time. Among all route combinations, C5 and P3A_03 exhibit the highest positive correlation. (2) A one-way risk spillover exists between P1A_03 an C5, while two-way positive risk spillover is observed between other routes. This suggests that when a risk materializes on a specific route, other routes are also exposed to potential risks, with varying intensities of spillover. (3) The distance and geographical location of routes may be key factors influencing the differing intensities of risk spillover. This highlights the need to consider the geographical characteristics of routes in understanding risk transmission. This paper aims to provide risk management strategies based on these empirical findings, assisting shipping companies and investors in developing more effective responses to market volatility.

## Section 1: Introduction

The influence of the shipping market on the world economy and international trade is increasingly significant. Over 80% of global trade is now transported by shipping [1]. The international dry bulk shipping market is a crucial component of the broader shipping industry, characterized by a high degree of maturity and route segmentation. This market is divided into four sub-markets based on ship tonnage, with each sub-market comprising multiple routes, each having its own freight rate.

The Baltic Exchange Dry Index (BDI) aggregates the freight rate indices of various ship types, reflecting the level of activity in shipping and trade. It effectively captures the current state of the global shipping market and the dynamics of international trade. Often regarded as a leading indicator of future economic growth or contraction, the BDI is frequently referred to as a “barometer” of the global economy.

**Fig 1:**
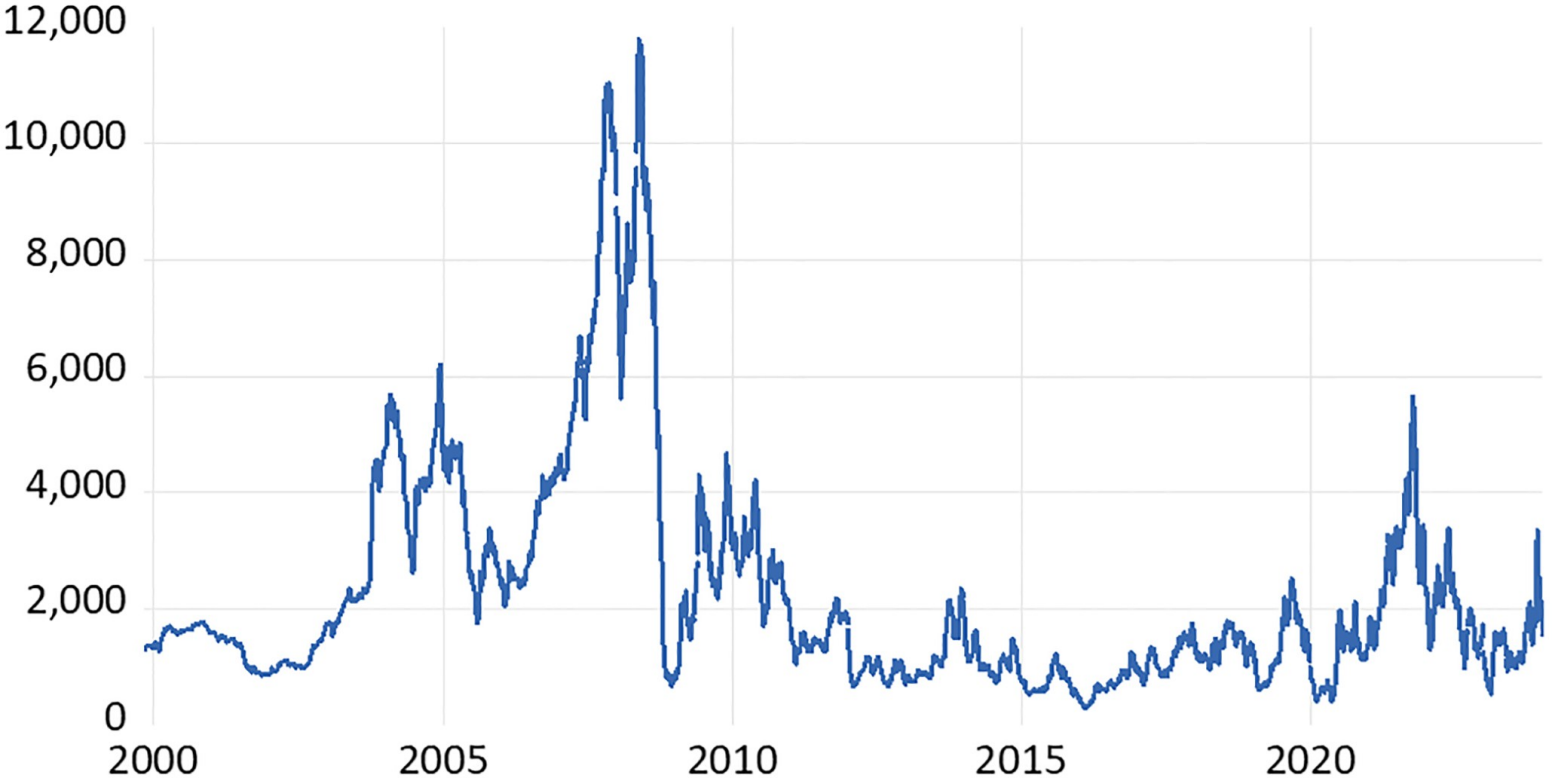
Trend plots of BDI diurnal data from 1999 to 2023.

With the advent of global economic flows and the high-speed information age, dry bulk freight rates have experienced significant fluctuations over the years due to various external factors. These include changes in the world economy, geopolitical events, fluctuations in raw material prices, and extreme occurrences. As shown in Fig 1, the BDI exhibits clear time-varying characteristics. The average BDI was 1,338.05 points in 1999, compared to 1,250.69 points in 2023. Following sharp fluctuations during the 2008 financial crisis, the BDI market experienced a substantial decline. Prior to 2008, the average freight rate was consistently higher than in the subsequent decade, and post-2008, rates have continued to fluctuate. Additionally, the global COVID-19 pandemic in 2020 and the Russia-Ukraine conflict in 2022 have led to significant changes in the BDI market.

**Fig 2:**
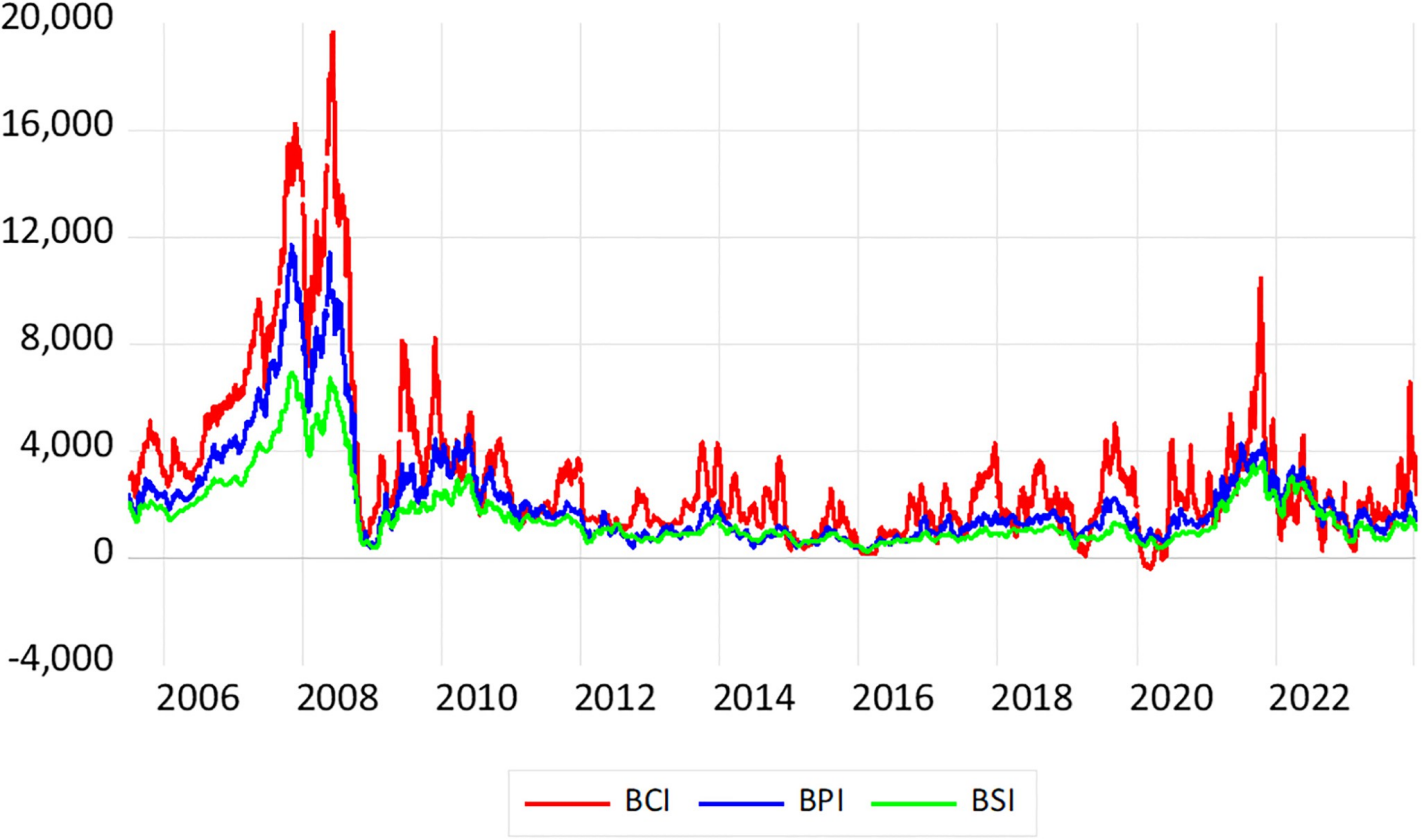
Trend plots of three dry bulk freight rates.

The BDI is a weighted composite index comprising the Baltic Capesize Index (BCI), Baltic Panamax Index (BPI), and Baltic Supramax Index (BSI). These three indices represent the freight rates for different classes of dry bulk carriers. As illustrated in Fig 2, the trends in BCI, BPI, and BSI over the years are generally aligned. Significant events such as the financial crisis, the global COVID-19 pandemic, and various international conflicts—including the China-US trade war and the Russia-Ukraine war—have caused substantial turbulence in the dry bulk market.

The BCI and BPI markets are widely regarded as leading indicators of global economic activity. Its fluctuations not only reflect supply and demand in the shipping market, but also indicate the impact of economic growth, trade policy and geopolitics. For shipping companies, shippers and investors, understanding changes in BCI and BPI is essential to optimize resource allocation, develop transportation strategies and assess investment risk. Effectively grasping the dynamic changes of these indicators can help enterprises gain advantages in the competitive market. As the complexity of global trade increases, the shipping market faces multiple risks such as price volatility, policy changes and environmental regulations. An in-depth study of BCI and BPI can provide industry players with data-driven risk management tools and investment decision support.

In this paper, we establish a comprehensive methodological framework that integrates the GARCH model, Copula function, and CoVaR model, which not only accounts for the volatility of time series but also captures the non-linear dependencies between routes, enabling a more accurate assessment of risk spillovers. The main contributions of this paper are as follows: (1) We effectively capture the volatility characteristics of freight rates for each route within the BCI and BPI markets by utilizing the time-varying coefficient features of the GARCH model. (2) The Copula method allows for a flexible capture of the dynamic dependency structure between the BCI and BPI markets, providing a more precise reflection of their nonlinear and heteroscedastic relationships compared to traditional linear correlation analyses. (3) We quantify the risk associated with individual routes and, in extreme cases, analyze the risk impact of one route on another using the CoVaR model. This two-way risk spillover analysis reveals the interdependence between markets, aiding shipping companies and investors in understanding the risk transmission mechanisms during market fluctuations or crises. It offers essential risk management insights and helps formulate more effective strategies in response to market volatility.

This paper is carried out under the following hypothesis:

*Hypothesis 1*: Dry bulk freight index has significant volatility, and its volatility is affected by economic and market factors.
*Hypothesis 2*: There is significant risk spillover effect between the dry bulk freight index.
*Hypothesis 3*: The GARCH-Copula-CoVaR model can effectively capture the volatility characteristics and risk spillover relationship of dry bulk freight index, which is better than the traditional model.

The remainder of this paper is organized as follows. The literature review is given in Section 2. The hybrid GARCH-Copula-CoVaR model are shown in Section 3. We describe the data in Section 4. Section 5 demonstrate the empirical results. In Section 6, we further discuss the risk spillover between route rates. In addition, Section 7 gives the concluding comments and some suggestions.

## Section 2: Literature review

### Research on volatility and correlation of dry bulk market

Studies examining the fluctuations in dry bulk markets often fall into two distinct categories. The first focuses on the volatility and correlation between the dry bulk market and other markets, while the second investigates the volatility and correlation within the dry bulk market itself. For instance, [2] utilized non-parametric causal quantiles to analyze the asymmetric relationship between the BDI and the spot prices of bulk commodities, finding that fluctuations in commodity prices can lead to spillovers in BDI volatility. However, the impact of the BDI on commodity prices varies significantly depending on the type of commodity and prevailing market conditions. For example, [3] identified a notable correlation between the BDI and crude oil prices, observing that this relationship is strong in the short term but weakens over the long term. Similarly, [4] employed Granger causality tests and co-integration analysis to explore the leading-lag relationships among the BDI, the Shanghai Container Freight Index (SCFI), and the Baltic Dirty Tanker Index (BDTI). They also applied multivariate impulse response functions and variance decomposition to assess how the freight market reacts to shocks in other freight markets. Their findings indicate that the dry bulk market is influenced by fluctuations in both the container and tanker shipping markets, with mutual volatility conduction observed specifically between the dry bulk and container shipping markets.

There is a substantial body of literature examining the volatility correlations within the primary dry bulk market. For example, [5] employed a hybrid model combining wavelet analysis and neural networks to investigate the fluctuations of freight indices for the 2A and 3A routes in the BPI market. Their time-series wavelet multi-scale decomposition highlighted the dynamics of fluctuations across different time frequencies. Similarly, [6] utilized the multifractal detrending volatility analysis (MD-DFA) technique to analyze market trends in the dry bulk freight indices of Capesize and Panamax vessels. Furthermore, [7] demonstrated that the contribution of the BPI market to the BDI has gradually increased, underscoring the significance of the Panamax bulk carrier market and its considerable influence on the development of the BDI. In another study, [8] applied Rescaled Range Analysis (R/S) and an enhanced R/S analysis method to examine the long-term memory of two shipping submarkets based on the freight indices of Panamax and Handysize vessels. While there have been numerous achievements regarding the fluctuation correlations between dry bulk markets and external factors, as well as within the internal primary market, there remains a notable gap in research focusing on the volatility and correlation of freight rates across various routes within the internal dry bulk markets.

### Research on risk spillovers of international dry bulk market

The sub-segments of the dry bulk shipping market are interconnected, and previous studies have highlighted the spillover effects among these sub-segments. There are differences in the response of dry bulk shipping market sub-segments to shocks [9]. There are some studies on risk spillovers between dry bulk market segments or with other markets. For instance, [10] was the first to combine long memory processes with Value at Risk (VaR) to examine risk spillover within the international dry bulk shipping market. Additionally, [11] utilized a three-variable VAR-BEKK-GARCH-X model to analyze the spillover effects between the BDI and the financial markets, and find that these spillover effects are time-varying and become more pronounced during the 2008–2009 global financial crisis. Furthermore, [12] employed a GARCH-Copula-CoVaR approach to investigate the extreme risk spillovers from the commodity market to the maritime sector, taking into account the interactions among different sub-sectors of the maritime market. Meanwhile, [13] explored risk measures, risk attitudes, and variable control related to the freight rate cycle in the dry bulk shipping market across various scenarios, concluding that there is a negative correlation between risk and return in long-term contracts. In addition, The dry bulk industry and the oil market often study their risk spillovers together. Compared with the dry bulk market, the oil tanker market has a higher integration degree, the high spillover period lasts longer, and the oil price fluctuation contributes more to the spillover effect of the oil tanker market [14]. Despite these contributions, there is a notable gap in the literature regarding risk spillover between routes within the dry bulk shipping market. Therefore, this paper aims to address this gap, providing valuable data support for the market planning of relevant shipping enterprises.

### Research on empirical methodology

Compared to the ARCH model, the GARCH model offers a linear extension of variance representation, effectively addressing the computational inefficiencies and accuracy limitations associated with high-order ARCH models. Given the GARCH family’s capacity to fit marginal distributions, it is particularly suitable for analyzing the volatility of freight rates in the dry bulk market [15]. [16] introduced the Copula function, which posits that the joint distribution of multivariate variables can be expressed as a function that combines the marginal probability distributions of each variable with a description of the correlation structure among them. The Copula function has found widespread application across various fields ([16, 17]). VaR is the most commonly used method for measuring the risk of individual institutions. However, it may not adequately reflect systemic risk, especially during periods of market instability. To address this gap, [18] introduced tail correlation analysis and proposed the Conditional Value at Risk (CoVaR) method. The Copula function is employed to calculate CoVaR ([19–21]).

The hybrid GARCH-Copula-CoVaR method has gained significant traction in modeling volatility correlations and risk spillovers across financial markets. For instance, [22] employed various GARCH-Copula models to analyze the tail dependencies among oil prices, investor expectations, and stock returns. Similarly, [23] introduced a GARCH-Copula deformation model to assess whether gold, the US dollar, and Bitcoin serve as hedging or safe-haven assets for stocks, and their potential in diversifying downside risks in international stock markets. Recently, this model has been extended to other fields, such as energy; [24] utilized the Copula framework to uncover the nonlinear tail-dependent structures between carbon and energy markets, calculating CoVaR to quantify extreme risk spillover effects. Findings reveal that during extreme events, risk spillovers from both traditional and renewable energy markets to carbon markets significantly increase. The GARCH-copula regression model is also used to analyze the heterogeneity of dynamic risk spillovers between logistics market and e-commerce market [25]. In the maritime sector, the GARCH-Copula-CoVaR approach offered new insights into risk transmission from oil and energy markets to maritime markets, highlighting interactions among various subsectors within the maritime industry [26].

Unlike [26], which focused on the risk spillover effects between oil, the ex-energy sector, and the BDI market, this paper investigates the volatility, correlation, and risk spillover effects among the main routes of the dry bulk shipping sub-market. Specifically, when the freight rate for a particular route experiences a sharp rise or fall, the tail dependence structure between that route and others is non-linear. To explore this phenomenon, we calculate the CoVaR using a static GARCH-Copula approach to examine risk spillover between routes. Additionally, we employ time-varying copula functions to assess the dynamic and tail correlations between the BCI and the BPI markets. Given that declines in freight rates typically result in greater losses, this paper primarily focuses on risk spillover during periods of market decline.

## Section 3: Methodology

### Marginal distribution model

The GARCH model effectively captures the time-varying volatility of financial time series, reflecting market volatility characteristics across different periods, which is essential for understanding and predicting financial risk. To account for the effects of autocorrelation and positive and negative shocks on conditional fluctuations, researchers often integrate an ARMA(r,s) model with the GARCH(p,q) framework. In this paper, we utilize the ARMA(r,s)-GARCH(p,q) model to analyze the time series of freight rates and derive the edge distribution.

$$\begin{array}{c} \left\{ \begin{array}{cc} r_{t}= & \mu +\sum_{i=1}^{r}\varphi_{i}r_{t-i}+\sum_{j=1}^{s}\theta_{j}a_{t-j}+a_{t},\\ a_{t}= & \sigma_{t}\epsilon_{t},\\ \sigma_{t}^{2}= & \omega +\sum_{i=1}^{p}\alpha_{i}a_{t-i}^{2}+\sum_{j=1}^{q}\beta_{j}\sigma_{t-j}^{2} \end{array}\right. \end{array}$$ (1)

The first line of Eq (1) represents the mean value equation of the ARMA(r,s) model, while the third line denotes the conditional variance equation. Here, *γ* is the maximum lag order of the autoregressive term, which influences the complexity of the autoregressive component, and *s* is the maximum lag order of the moving average, determining its impact on the model. *μ* is the constant term, *φ*_*i*_ is the coefficient of AR(r), *θ*_*j*_ is the coefficient of MA(s), and *ω* is the mean of conditional variance regression term. *a*_*t*_ is the residual term. Since the freight rates collected in this paper has a heavier tail, ARMA(r,s)-GARCH(p,q) under the skew-t distribution is selected. *σ*_*t*_ denotes the conditional standard deviation, which measures return volatility, while $\sigma_{t}^{2}$ indicates the conditional variance, capturing the squared effects of these fluctuations. The perturbation term {*ϵ*_*t*_} is typically assumed to be an independent and identically distributed sequence of random variables with a mean of 0 and a variance of 1.

### Static and dynamic Copula functions

The Copula method is able to model dependencies between multiple variables, especially in cases where the data does not conform to a normal distribution. It captures tail dependencies between financial assets, which is particularly important during periods of market turmoil. When the random variables with different edge distributions are not independent, the traditional joint distribution fitting method is inadequate. To solve this puzzle, the Copula function can be used to fit the joint distribution accurately and flexibly for random variables with multiple edge distributions.

Binary Sklar’s theorem: Let *H*(⋅, ⋅) be a joint distribution function with edge distributions *F*(⋅) and *G*(⋅), then there is a function *C*(⋅, ⋅) such that:

$$\begin{array}{c} H(x,y)=C(F(x),G(y)), \end{array}$$ (2)

where *C*(⋅, ⋅) is corresponding Copula function. The original function *H*(*x*, *y*) can be written as a function *C*(⋅, ⋅) related on *u* and *v*, then the function *C*(⋅, ⋅) is the Copula function, that is,

$$\begin{array}{c} C\left(u,v\right)=H(F^{-1}\left(u\right),G^{-1}\left(v\right)). \end{array}$$ (3)

To characterize the properties of freight rates exhibiting peak and thick tails, we introduce six static copula functions to describe the nonlinear relationships and tail correlations. Additionally, we incorporate three time-varying copula functions to capture the dynamic changes in correlation between the freight rates in the BCI and BPI markets. The formulas for these copula functions are presented in Table 1.

**Table 1:** Static and dynamic Copula functions.

Copula	Formula	Parameter
Static/Normal Copula	$C\left(u,v\right)=\int_{-\infty }^{\Phi^{-1}\left(u\right)}\int_{-\infty }^{\Phi^{-1}\left(v\right)}\frac{1}{2\pi \sqrt{1-\rho^{2}}}\text{exp}(\frac{-(r^{2}+s^{2}-2\rho rs)}{2(1-\rho^{2})})drds$	*ρ* ∈ (−1, 1)
*t* Copula	$C\left(u,v;\rho ,\kappa \right)=\int_{-\infty }^{T_{\kappa }^{-1}\left(u\right)}\int_{-\infty }^{T_{\kappa }^{-1}\left(v\right)}\frac{1}{2\pi \sqrt{1-\rho^{2}}}{\left[1+\frac{\left(t^{2}+s^{2}-2\rho ts\right)}{\kappa (1-\rho^{2})}\right]}^{-\frac{\kappa +2}{2}}dsdt$	*ρ* ∈ (−1.1)
Gumbel Copula	$C_{G}\left(u,v;a\right)=\text{exp}(-{\left[{\left(-lnu\right)}^{\frac{1}{a}}+{\left(-lnv\right)}^{\frac{1}{a}}\right]}^{a})$	*a* ∈ (0, 1]
Clayton Copula	$C_{CL}\left(u,v;\eta \right)={\left(u^{-\eta }+v^{-\eta }-1\right)}^{-\frac{1}{\eta }},$	*η* ∈ (0, ∞)
Frank Copula	$C_{F}\left(u,v;\lambda \right)=-\frac{1}{\lambda }ln(1+\frac{\left(e^{-\lambda u}-1\right)\left(e^{-\lambda v}-1\right)}{e^{-\lambda }-1})$	λ ≠ 0
SJC Copula	$C_{SJC}\left(u,v;\theta ,\delta \right)=1-{\left\{1-\text{exp}(-{\left[m(u)+m(v)\right]}^{\frac{1}{\delta }})\right\}}^{\frac{1}{\theta }}$	*θ* ≥ 1, *δ* > 0
Dynamic/Normal Copula	$C\left(u,v\right)=\int_{-\infty }^{\Phi^{-1}\left(u\right)}\int_{-\infty }^{\Phi^{-1}\left(v\right)}\frac{1}{2\pi \sqrt{1-\rho_{t}^{2}}}\text{exp}(\frac{-(r^{2}+s^{2}-2\rho_{t}rs)}{2(1-\rho_{t}^{2})})drds$	*ρ*_*t*_ ∈ (−1, 1)
SJC Copula	$C_{SJC}\left(u,v|\tau^{U},\tau^{L}\right)=\frac{1}{2}(C_{JC}(u,v|\tau^{U},\tau^{L})+C_{JC}(1-u,1-v|\tau^{U},\tau^{L})+u+v-1)$	*τ*^*U*^, *τ*^*L*^ ∈ (0, 1)
Rotated Gumbel Copula	$C_{RG}\left(u,v;\rho_{t}\right)=\text{exp}\{-{\left\{-{\left[k\left(u\right)\right]}^{\rho_{t}}+\left[-k\left(u\right)\rho_{t}\right]\right\}}^{1/\rho_{t}}\}{\left(l\left(u\right)+l\left(v\right)-1\right)}^{-1/\theta }$	*θ* ∈ (0, ∞)

Note: Φ(⋅) is normal distribution function. *T*_*κ*_(⋅) is *t* distribution function with degree of freedom *κ*.

*C*_*JC*_ is the distribution function of the time-varying Joe-Copula function. $\tilde{\Lambda }=\left(1-e^{-x}\right)/\left(1+e^{-x}\right)$,

*m*(*u*) = (−ln(1 − (1 − *u*)^*θ*^))^*δ*^, *m*(*v*) = (−ln(1 − (1 − *v*)^*θ*^))^*δ*^, *k*(*u*) = ln(1 − *u*), *k*(*v*) = ln(1 − *v*),

*l*(*u*) = (1 − *u*)^−*θ*^ and *l*(*v*) = (1 − *v*)^−*θ*^.

### CoVaR methodology

1. *CoVaR*. VaR is primarily used to assess the risk associated with a specific asset, but it fails to capture systemic risk within a market. Additionally, VaR is limited in its ability to measure risks that arise under extreme conditions. Therefore, this paper considers the CoVaR approach, which builds on the foundation of VaR. The CoVaR model measures the potential losses of one route when a risk materializes in another route, making it particularly valuable for analyzing financial system stability and assessing systemic risk. For example, the extreme loss $CoVaR_{\alpha ,\beta ,t}^{s|i}$ of the freight return *Y*_*s*,*t*_ occurs under the condition that the loss $VaR_{\alpha ,t}^{i}$ is defined as follows:
  $$\begin{array}{c} P(Y_{s,t}\leq CoVaR_{a^{d},\beta^{d},t}^{s|i,D}|Y_{i,t}=VaR_{a^{d},t}^{i,D})=\beta^{d}. \end{array}$$ (4)
  CoVaR can be divided into ascending CoVaR and descending CoVaR according to different confidence levels. $CoVaR_{a^{d},\beta^{d},t}^{s|i,D}$ above is the descending condition at risk value of *Y*_*s*,*t*_ when *Y*_*i*,*t*_ is under the condition of descending risk. The corresponding ascending CoVaR expression is as follows:
  $$\begin{array}{c} P(Y_{s,t}\geq CoVaR_{a^{u},\beta^{u},t}^{s|i,U}|Y_{i,t}=VaR_{a^{u},t}^{i,U})=\beta^{u}, \end{array}$$ (5)
  where *β*^*d*^ + *β*^*u*^ = 1, *β*^*u*^ = 0.05 and *β*^*d*^ = 0.95.
2. Δ*CoVaR*. Whether $CoVaR_{a^{d},\beta^{d},t}^{s|i,D}$ or $CoVaR_{a^{u},\beta^{u},t}^{s|i,U}$ they still included in the calculation of VaR, can not measure spillover effect between the two routes. Therefore, ΔCoVaR is usually used to measure the risk spillover effect between different freight rates of various routes. For example, the downside risk spillover of the freight return for *s* route under the freight return of *i* route is expressed as follows:
  $$\begin{array}{c} \Delta CoVaR_{a^{d},\beta^{d},t}^{s|i,D}=CoVaR_{a^{d},\beta^{d},t}^{s|i,D}-VaR_{a^{d},t}^{s,D}. \end{array}$$ (6)
  Correspondingly, the downside risk spillover of the freight rate for *i* route under the freight rate return of *s* route is as follows:
  $$\begin{array}{c} \Delta CoVaR_{a^{d},\beta^{d},t}^{i|s,D}=CoVaR_{a^{d},\beta^{d},t}^{i|s,D}-VaR_{a^{d},t}^{i,D}. \end{array}$$ (7)
3. *%CoVaR*. Although ΔCoVaR has been calculated to measure the risk spillover effect, the %CoVaR is continued to be considered to remove the dimensional effect. For example, the %CoVaR of the freight rate return for *s* route to the freight rate return of *i* route is as follows:
  $$\begin{array}{c} \%CoVaR=\frac{CoVaR_{a^{d},\beta^{d},t}^{i|s,D}}{VaR_{a^{d},t}^{i,D}}\times 100\%. \end{array}$$ (8)
  %CoVaR represents a relative change, and the %CoVaR rank is used to compare the risk spillover degree between different routes. Since the extreme loss mainly occurs in the period of economic downturn, we mainly studies the risk spillover effect in the downward state, and so ΔCoVaR and %CoVaR are.

### GARCH-Copula-CoVaR model

The hybrid Garch-Copula-CoVaR model used the GARCH model to describe the marginal distribution of the freight return of each route. Set the marginal distribution of the freight return for the routes as *G*(*t*) and the edge density function as *g*(*t*). *c*(⋅, ⋅) is obtained from the first derivative of the optimal Copula, that is, $c\left(u,v\right)=\frac{\partial C(u,v)}{\partial u\partial v}$. The specific steps are as follows.

**Step 1**: (*X*, *Y*) are defined as two-dimensional continuous random variables. *f*(*x*) represents the edge distribution density function of *X*. *g*(*y*) represents the edge distribution density function of *Y*. *h*(*X*, *Y*) is defined as the joint density function of (*X*, *Y*). Then we have
  $$\begin{array}{c} P\left(Y\leq y|X=x\right)=lim_{h\to 0}P\left(Y\leq y|x\leq X\leq x+h\right)=\int_{-\infty }^{y}\frac{h(x,t)}{f(x)}dt. \end{array}$$ (9)
  From binary Sklar’s theorem, we can get
  $$\begin{array}{c} \int_{-\infty }^{y}\frac{h(x,t)}{f(x)}dt=\int_{-\infty }^{y}\frac{c(F(x),G(t))f(x)g(t)}{f(x)}dt=\int_{-\infty }^{y}c\left(F\left(x\right),G\left(t\right)\right)g\left(t\right)dt. \end{array}$$ (10)
**Step 2**: Substitute (10) into (4), then one can obatin
  $$\begin{array}{c} P(Y_{s,t}\leq CoVaR_{a^{d},\beta^{d},t}^{s|i,D}|Y_{i,t}=VaR_{a^{d},t}^{i,D})=\int_{-\infty }^{Y_{s,t}\leq CoVaR_{a^{d},\beta^{d},t}^{s|i,D}}c\left(F\left(VaR_{a^{d},t}^{i,D}\right),G\left(t\right)\right)g\left(t\right)dt. \end{array}$$ (11)
**Step 3**: Then we achieve
  $$\begin{array}{c} P(Y_{s,t}\leq CoVaR_{a^{d},\beta^{d},t}^{s|i,D}|Y_{i,t}=VaR_{a^{d},t}^{i,D})=\int_{-\infty }^{Y_{s,t}\leq CoVaR_{a^{d},\beta^{d},t}^{s|i,D}}c\left(a^{d},G\left(t\right)\right)g\left(t\right)dt=\beta^{d}. \end{array}$$ (12)
  Hence, $CoVaR_{a^{d},\beta^{d},t}^{s|i,D}$ can be calculated by inverse solution method. the $\Delta CoVaR_{a^{d},\beta^{d},t}^{s|i,D}$ and $\%CoVaR_{a^{d},\beta^{d},t}^{s|i,D}$ can be get by (7) and (8), respectively.

## Section 4: Data

### Data source

We selected the daily freight rates for the main routes in the BCI and BDI markets, which are widely utilized in maritime economics research. The BCI serves as an index for the Capesize dry bulk market and is constructed based on the spot freight rates of Capesize dry bulk carriers, known for their substantial capacity in the dry bulk shipping sector. In the transportation of dry bulk cargo, iron ore represents the largest share of total sea freight. The C2, C3 and C5 routes are the three most significant pathways for iron ore transport, making them ideal representatives of the BCI market.

The BPI is an index that represents the Panamax dry bulk market, based on the spot freight rates of Panamax dry bulk carriers. These vessels play a crucial role in the dry bulk shipping sector due to their significant capacity. The P1A route connects the Americas to Western Europe, P2A links the Far East with the Atlantic Ocean, and P3A connects the Americas to the Far East. These routes are among the busiest in the world. Given that P1A_03, P2A_03 and P3A_03 are more established than other routes, they have been selected to represent the BPI market.

**Fig 3:**
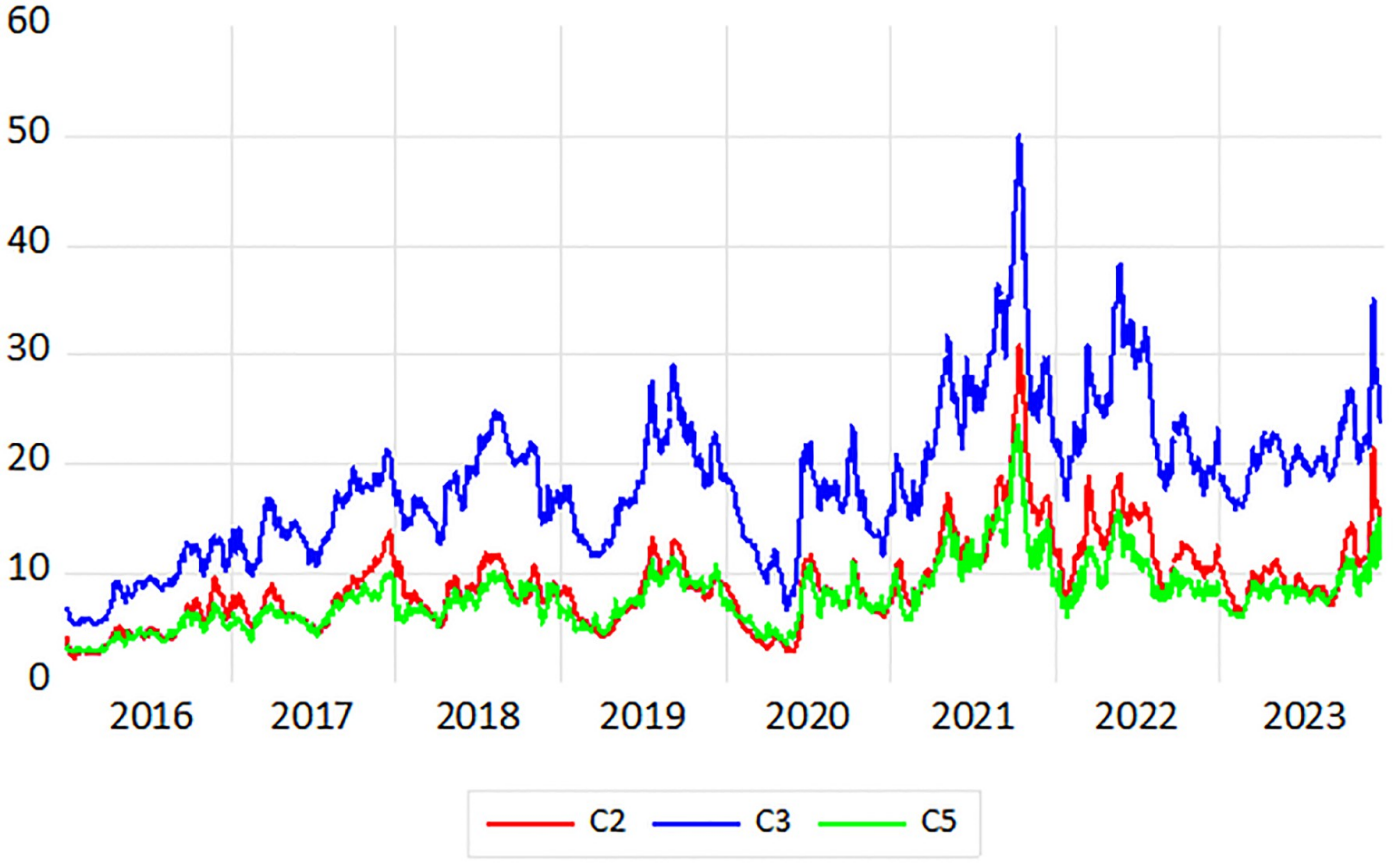
Trend plots of freight rates for C2, C3 and C5 routes.

**Fig 4:**
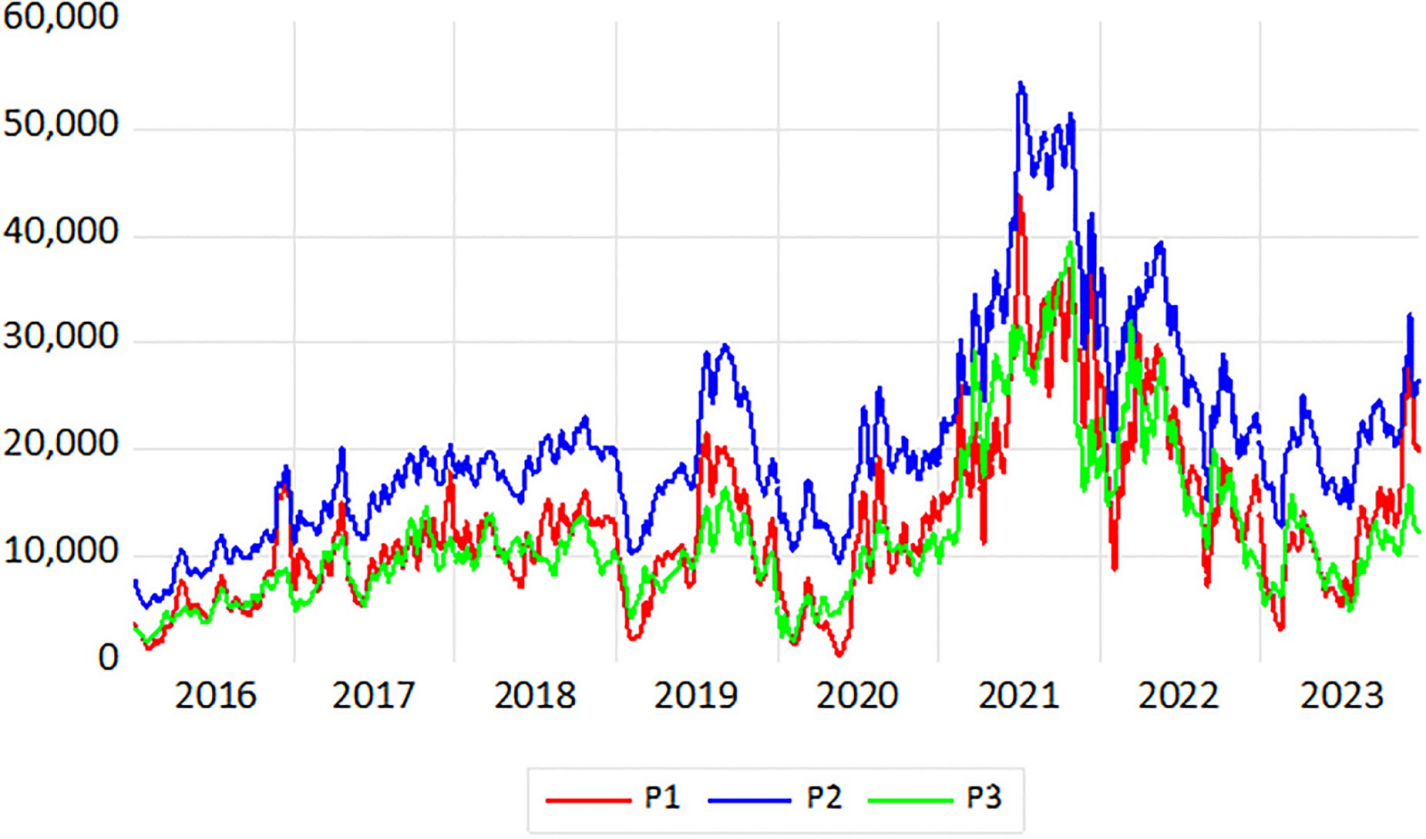
Trend plots of freight rates for P1, P2 and P3 routes.

This paper collects daily freight rates for six routes from January 4, 2016 to December 22, 2023. The Baltic Sea Exchange did not record transactions on non-working days, resulting in a total of 1,995 days and generating a dataset of 1, 995*6 = 11, 970 observations. The data were sourced from the Clarkson website (https://sin.clarksons.net/). The analysis was conducted using Eviews, R, and MATLAB. The current routes for Capesize and Panamax dry bulk carriers are presented in Table 2, while the time series plots of freight rates for the main routes in the BCI and BPI markets are illustrated in Figs 3 and 4.

**Table 2:** Capesize dry bulk carrier routes.

Sector	Short Description	Size(MT)	Price quotation	Time intervals
C2	Tubarao to Rotterdam	160000	USD/Tonne	4/1/2016 to 22/12/2023
C3	Tubarao to Qingdao	160000 /170000	USD/Tonne	4/1/2016 to 22/12/2023
C5	West Australia to Qingdao	160000	USD/Tonne	4/1/2016 to 22/12/2023
P1	Skaw-Gib transatlantic round voyage	74000	USD/Day	4/1/2016 to 22/12/2023
P2	Skaw-Gib trip HK-S Korea incl Taiwan	74000	USD/Day	4/1/2016 to 22/12/2023
P3	HK-S Korea incl Taiwan, one Pacific RV	74000	USD/Day	4/1/2016 to 22/12/2023

Note: P1, P2 and P3 represent P1A_03, P2A_03 and P3A_03, respectively.

### Data preprocessing

As shown in Figs 3 and 4, among the Capesize routes, the freight rate for the C3 route consistently exceeds that of the other two routes at various times, exhibiting similar fluctuation trends, particularly with significant volatility during the period from 2020 to 2022. For the Panamax routes, the freight rate for the P2 route is slightly higher than those of the other two routes, also demonstrating notable fluctuations during 2020 to 2022. Overall, the freight returns for all six routes display considerable instability. To assess the stationarity of the series, we calculate the returns using the logarithmic first-order difference as follows:

$$\begin{array}{c} Y_{t}=\left(lnP_{t}-lnP_{t-1}\right)\times 100, \end{array}$$ (13)

where *Y*_*t*_ represents the logarithmic return of route, and *P*_*t*_ represents the freight rate at time t. In order to increase the stability of the data, the unified magnification is 100 times. Denote C2*, C3*, C5*, P1*, P2*, P3* represent the logarithmic return of freight rate for C2, C3, C5, P1, P2 and P3 routes, respectively.

**Fig 5:**
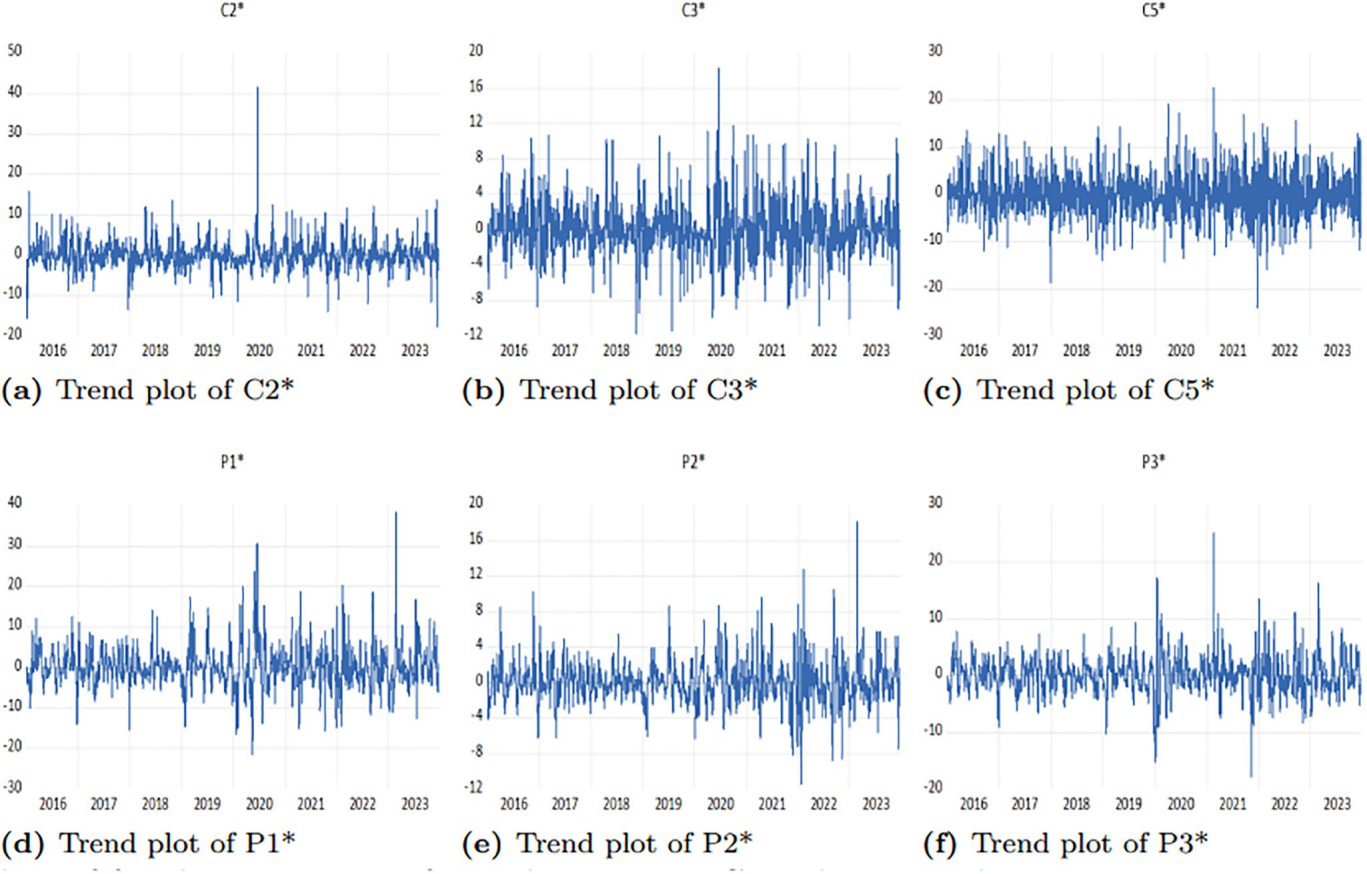
Trend plots of freight rate returns for each route in BCI and BPI markets.

Fig 5 displays the time series of logarithmic returns obtained by calculating the first-order difference of the freight rates. It is evident that the logarithmic returns for each route fluctuate around zero, showing similar patterns of volatility clustering coinciding with significant events such as the US-China trade disputes, the COVID-19 pandemic, and the Russia-Ukraine conflict. However, the responses of different routes to these extreme shocks have varied over time. These characteristics present an opportunity to explore risk spillovers between the freight rates in the BCI and BPI markets.

### Nonlinear analysis of returns series for each route

**Fig 6:**
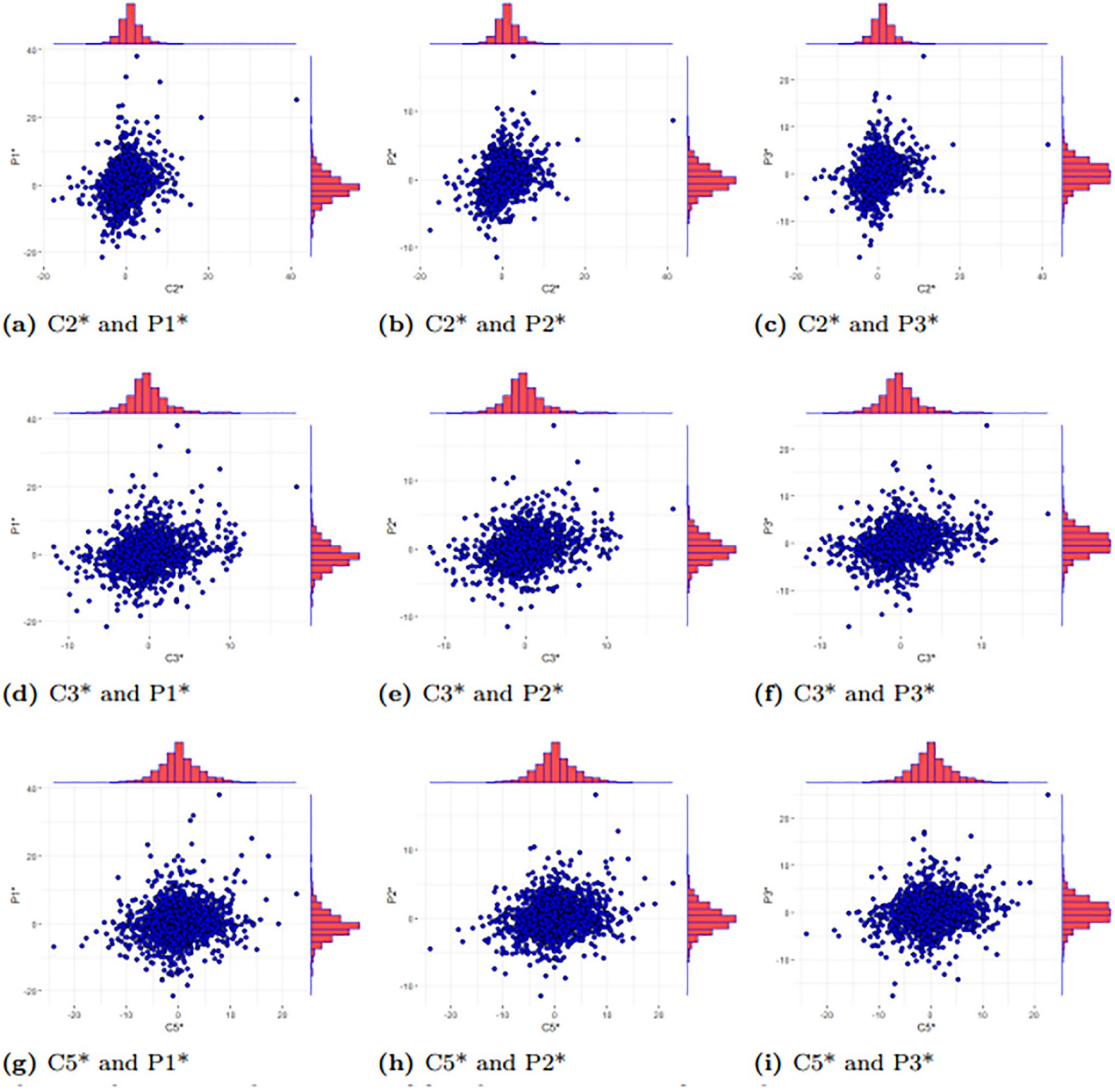
Scatter plots and sequence histogram of freight rate returns for each route.

Fig 6 presents scatter plots and histograms of the logarithmic returns for the various routes. The figure reveals a significant nonlinear correlation between the logarithmic returns of each route. As a result, traditional linear methods may not adequately capture the relationships between the routes. It is essential to employ a method that is suitable for nonlinear relationships in modeling. The Copula function effectively addresses linear constraints, facilitating research into nonlinear correlations.

### Descriptive statistics of returns series for each route

In Table 3, the mean logarithmic return for each route ranges from approximately 0.06 to 0.08, with a maximum value of 41.428 for C2* and a minimum value of -24.016 for C5*. The standard deviation for P1* is the highest, indicating significant fluctuations, while P2* has the lowest standard deviation, suggesting relatively stable fluctuations. The skewness values for all logarithmic returns are greater than zero, indicating that the distributions are not symmetrical and are skewed to the right. Additionally, the kurtosis values for all logarithmic returns exceed 3, signifying that these returns have higher peaks and thicker tails. The Jarque-Bera (J-B) test statistics are relatively large, and the corresponding *p*-values are less than 0.01, indicating that the logarithmic returns do not follow a normal distribution. The Augmented Dickey-Fuller (ADF) test results suggest that the sequences are stationary. Furthermore, the Q(12) test indicates no autocorrelation among the various return sequences at a significance level of 1% with a lag of 12 orders.

**Table 3:** Descriptive statistics of freight rate returns for each route (*p*-value).

Statistics	C2*	C3*	C5*	P1*	P2*	P3*
mean	0.065	0.067	0.063	0.086	0.062	0.068
median	-0.177	-0.105	-0.163	-0.142	-0.037	0.065
max	41.428	18.243	22.612	38.094	18.055	25.063
min	-17.584	-11.741	-24.015	-21.392	-11.433	-17.731
std	3.139	2.825	4.318	4.847	2.268	3.196
skew	1.503	0.558	0.148	0.894	0.599	0.350
kurtosis	21.233	6.330	5.146	8.749	7.203	7.732
J-B	28369.600	1024.876	389.746	3011.717	1587.041	1901.401
	(0.000)	(0.000)	(0.000)	(0.000)	(0.000)	(0.000)
ADF	-22.656	-24.818	-31.073	-13.451	-17.144	-15.774
	(0.000)	(0.000)	(0.000)	(0.000)	(0.000)	(0.000)
Q(12)	1217.705	666.603	260.680	2799.011	2403.303	2721.511
	(0.000)	(0.000)	(0.000)	(0.000)	(0.000)	(0.000)

Note: J-B stands for Jarque-Bera test statistic and is used to check whether the freight rate return follows a normal distribution.

### Endogeneity and heteroskedasticity tests

The Granger causality test helps identify the causal relationships between variables and assess endogeneity. As shown in Table 4, there is a one-way Granger causality from C2 to P2, C2 to P3, C3 to P2, C3 to P3, and C5 to P3 at a significance level of 10%. The remaining relationships exhibit two-way Granger causality. This indicates that the lagged values not only influence their own future outcomes but also provide statistically significant information to predict the outcomes of other markets, demonstrating a causal effect on other market sequences. These findings offer a crucial basis for understanding the linkages between freight rates across various routes.

**Table 4:** Results of Granger causality test.

Null hypothesis	F-statistics	p-value	conclusion
C2 is not the Granger cause of P1	2.306	0.0561	reject
P1 is not the Granger cause of C2	6.285	5.E-05	reject
C2 is not the Granger cause of P2	1.982	0.1592	no reject
P2 is not the Granger cause of C2	8.059	0.0046	reject
C2 is not the Granger cause of P3	0.741	0.4763	no reject
P3 is not the Granger cause of C2	2.659	0.0702	reject
C3 is not the Granger cause of P1	5.392	0.0003	reject
P1 is not the Granger cause of C3	2.353	0.0519	reject
C3 is not the Granger cause of P2	10.889	2.E-05	reject
P2 is not the Granger cause of C3	2.048	0.1292	no reject
C3 is not the Granger cause of P3	7.383	6.E-05	reject
P3 is not the Granger cause of C3	0.937	0.4219	no reject
C5 is not the Granger cause of P1	2.937	0.0532	reject
P1 is not the Granger cause of C5	5.569	0.0039	reject
C5 is not the Granger cause of P2	7.5412	0.0005	reject
P2 is not the Granger cause of C5	2.878	0.0565	reject
C5 is not the Granger cause of P3	6.869	0.0001	reject
P3 is not the Granger cause of C5	0.822	0.4814	norejet

The ARCH effect test is a crucial step in evaluating the heteroscedasticity of residuals after modeling high-frequency time series data. In this paper, we employ the LM test statistic to assess the ARCH effect. The results of the ARCH effect test for the residual variances of each sequence are presented in Table 5. As indicated by the *p*-values in the table, we reject the null hypothesis at a significance level of 0.05, confirming the presence of an ARCH effect in the logarithmic returns of each series. Each logarithmic return series exhibits characteristics such as sharp peaks, thick tails, non-normality, and conditional heteroscedasticity.

**Table 5:** Results of ARCH effect test.

Routes	LM test		Rank-based Test	
	test statistics	p-value	test statistics	p-value
C2*	374.328	0.000	63.178	0.000
C3*	69.502	0.000	154.954	0.000
C5*	107.118	0.000	73.861	0.000
P1*	211.283	0.000	228.929	0.000
P2*	445.453	0.000	175.134	0.000
P3*	462.863	0.000	153.479	0.000

## Section 5: Empirical analysis

### Edge distribution estimation

The primary purpose of using the GARCH model is to effectively capture the heteroscedasticity present in the logarithmic return series of freight rates across various routes, specifically the characteristics of volatility changes over time. When establishing a GARCH model, it is essential to make reasonable assumptions regarding the distribution of the residual perturbation terms in the series. Typically, residuals can be assumed to follow various distribution types, including the normal distribution, t-distribution, and skewed t-distribution. Therefore, we will examine the GARCH-normal model, GARCH-t model, and GARCH-skewed t model separately. We select the GARCH (p,q) order and residual distribution based on the AIC and Log Likelihood function (LLF), while passing the significance test of the parameters and considering the simplicity of the model.

Table 6 presents the GARCH(p,q) results for each sequence with various residual distributions, with the GARCH(1,1)-skew-t model ultimately selected to estimate the edge distribution. To mitigate the impact of a high-order mean model on subsequent modeling, ARMA(1,1) is consistently chosen under the constraint of sequence autocorrelation. Consequently, all sequences utilize the ARMA(1,1)-GARCH(1,1)-skew-t framework to characterize volatility.

**Table 6:** GARCH modeling and evaluation of freight rate returns for each route.

Distributions	C2*		C3*		C5*	
	AIC	LLF	AIC	LLF	AIC	LLF
GARCH(1,1)-t	4.284	-4266.247	4.252	-4234.021	5.4726	-5451.915
GARCH(1,1)-skew-t	4.279	-4261.042	4.248	-4228.982	5.4723	-5450.594
GARCH(1,1)-normal	4.511	-4493.621	4.446	-4427.454	5.5357	-5515.889
GARCH(2,1)-t	4.2849	-4266.201	4.253	-4233.963	5.4736	-5451.908
GARCH(2,1)-skew-t	4.281	-4260.976	4.249	-4228.905	5.4733	-5450.579
GARCH(2,1)-normal	4.512	-4493.423	4.445	-4427.334	5.5368	-5515.955
GARCH(1,2)-t	4.285	-4266.178	4.246	-4227.452	5.4713	-5449.653
GARCH(1,2)-skew-t	4.281	-4260.912	4.242	-4221.981	5.4710	-5448.276
GARCH(1,2)-normal	4.511	-4492.907	4.443	-4425.312	5.5349	-5514.054
GARCH(2,2)-t	4.285	-4266.178	4.247	-4227.452	5.4723	-5449.653
GARCH(2,2)-skew-t	4.282	-4260.912	4.243	-4221.981	5.4720	-5448.276
GARCH(2,2)-normal	4.512	-4492.907	4.444	-4425.312	5.5359	-5514.054
Distributions	P1*		P2*		P3*	
	AIC	LLF	AIC	LLF	AIC	LLF
GARCH(1,1)-t	4.333	-4314.974	2.922	-2907.477	3.434	-3418.228
GARCH(1,1)-skew-t	4.332	-4313.248	2.915	-2899.983	3.425	-3407.889
GARCH(1,1)-normal	4.545	-4527.205	3.092	-3077.744	3.587	-3572.459
GARCH(2,1)-t	4.334	-4315.142	2.923	-2907.144	3.435	-3418.452
GARCH(2,1)-skew-t	4.333	-4313.394	2.916	-2899.614	3.426	-3408.107
GARCH(2,1)-normal	4.546	-4527.491	3.092	-3077.619	3.589	-3572.840
GARCH(1,2)-t	4.332	-4313.551	2.921	-2905.358	3.434	-3417.721
GARCH(1,2)-skew-t	4.332	-4311.924	2.915	-2898.328	3.425	-3407.475
GARCH(1,2)-normal	4.536	-4517.588	3.092	-3077.255	3.586	-3569.666
GARCH(2,2)-t	4.285	-4312.074	2.922	-2905.358	3.435	-3417.721
GARCH(2,2)-skew-t	4.331	-4310.503	2.916	-2898.328	3.426	-3407.475
GARCH(2,2)-normal	4.537	-4517.437	3.093	-3077.255	3.587	-3569.666

In Table 7, all values of *α*_1_ + *β*_1_ are less than 1, which ensures the effectiveness of the GARCH model in fitting the fluctuations of freight rates for the internal routes in BCI and BPI markets. Except for C2* series, all other *β*_1_ is greater than the corresponding *α*_1_, which indicates that they all have strong and sustained fluctuations during the sample period. The values of *α*_1_ + *β*_1_ for C3* series and P1* series are very close to 1, which indicates that these two returns series have higher sustained volatility and long memory than other series. The values of shape range from 2 to 5, and all *p*-values are very close to 0, indicating that the degree of freedom parameter is suitable for data with significant skewness and thick tails. The *p*-values of ARCH-LM test are all greater than 0.1, which indicates that the ARCH effect no longer exists in these series. That is, the GARCH(1,1)-Skew-t model eliminates the ARCH effect in each series (ARCH-LM test uses 10th order). Overall, the model and edge distribution selected here are appropriate.

**Table 7:** ARMA(1,1)-GARCH(1,1)-skew-t modeling parameters (*p*-value).

Parameters	C2*	C3*	C5*	P1*	P2*	P3*
*μ*	0.017	-0.019	0.001	-0.2417	0.050	0.102
	(0.886)	(0.830)	(0.996)	(0.282)	(0.684)	(0.583)
*φ*_1_	0.555	0.364	0.030	0.764	0.772	0.796
	(0.000)	(0.000)	(0.542)	(0.000)	(0.000)	(0.000)
*θ*_1_	0.089	0.255	0.371	0.225	0.241	0.307
	(0.009)	(0.000)	(0.000)	(0.000)	(0.000)	(0.000)
*ω*	3.312	0.462	1.408	0.791	0.263	0.384
	(0.0000)	(0.053)	(0.003)	(0.000)	(0.000)	(0.000)
*α*_1_	0.428	0.263	0.170	0.436	0.446	0.373
	(0.000)	(0.000)	(0.000)	(0.000)	(0.000)	(0.000)
*β*_1_	0.221	0.736	0.766	0.563	0.503	0.542
	(0.018)	(0.000)	(0.000)	(0.000)	(0.000)	(0.000)
skew	1.104	1.096	1.048	1.058	1.125	1.154
	(0.000)	(0.000)	(0.000)	(0.000)	(0.000)	(0.000)
shape	2.993	3.114	4.747	3.4097	3.594	4.123
	(0.000)	(0.000)	(0.000)	(0.000)	(0.000)	(0.000)
ARCH	8.795	11.952	9.490	10.311	10.546	10.257
	(0.552)	(0.288)	(0.486)	(0.414)	(0.394)	(0.418)

Note: *μ* is a constant term of the mean. *φ*_1_ and *θ*_1_ represents the coefficients of the mean equation, except for C5* series, all other returns series have passed the test. *ω* is the constant term of conditional variance, except for C3* series, all other returns series have passed the test. *α*_1_ is the ARCH(1) coefficient, which reflects the magnitude of the fluctuation. *β*_1_ is the GARCH(1,1) coefficient, which reflects the duration of the fluctuation. Skew is a skewness parameter. Shape is a degree of freedom parameter.

### Nonlinear correlation analysis

#### Static Copula correlation analysis

After estimating the GARCH edge distribution, we can obtain the conditional mean, conditional standard deviation, and standardized residuals of the sequence. The correlation between paired series will be derived from the probability integral transformation in the BCI and BPI markets using binary Copula modeling. The most suitable Copula function is selected based on the criterion of minimizing the Akaike Information Criterion (AIC).

In Table 8, all pairs, except for C2*-P2*, C3*-P2*, and C5*-P1*, utilize the t Copula as the optimal function, indicating a degree of homogeneity among these routes. The correlations for C2*-P1* and C3*-P1* are relatively low, which may be attributed to the impact of route locations on their correlation. The P1 route connects Europe and America along the west coast of the Atlantic, while the C2 route links Brazil with Western Europe, and the C3 route connects Brazil to China. With the exceptions of C3*-P2* and C5*-P1*, there is a symmetric positive correlation in the tails of all other routes. Overall, a positive correlation exists among the various routes.

**Table 8:** Copula functions modeling parameters.

routes	Normal Copula				t Copula						
	LLF	AIC	$\hat{\rho }$	K-*τ*	LLF	AIC	$\hat{\rho }$	$\hat{\kappa }$	K-*τ*	UTD	LTD
C2*-P1*	31.66	-61.33	0.17	0.11	37.04	-70.07	0.17	14.03	0.11	0.0053	0.0053
C2*-P2*	51.22	-100.43	0.21	0.13	50.68	-107.35	0.21	14.74	0.14	0.0057	0.0057
C2*-P3*	52.03	-102.06	0.22	0.14	54.21	-104.43	0.22	22.01	0.14	0.008	0.008
C3*-P1*	34.32	-66.64	0.17	0.11	39.03	-74.05	0.18	14.74	0.11	0.0044	0.0044
C3*-P2*	48.77	-95.54	0.21	0.13	49.50	-95.00	0.21	30	0.13	0	0
C3*-P3*	59.54	-117.09	0.23	0.15	61.61	-119.22	0.23	21.33	0.15	0.0011	0.0011
C5*-P1*	28.89	-55.78	0.16	0.1	29.7	-55.41	0.17	30 0.11	0	0	
C5*-P2*	39.5	-76.99	0.19	0.12	40.95	-77.89	0.19	26.2	0.12	0.002	0.002
C5*-P3*	62.02	-122.05	0.24	0.16	66.01	-128.02	0.24	17.24	0.16	0.0037	0.0037
routes	Frank Copula				SJC Copula						
	LLF	AIC	$\hat{\lambda }$	K-*τ*	LLF	AIC	$\hat{\theta }$	$\hat{\delta }$	K-*τ*	UTD	LTD
C2*-P1*	32.93	-63.87	1.07	0.12	33.53	-63.05	1.06	0.16	0.1	0.07	0.01
C2*-P2*	46.53	-91.06	1.27	0.14	56.41	-108.81	1.07	0.22	0.13	0.09	0.04
C2*-P3*	47.53	-93.06	1.29	0.14	50.48	-96.96	1.08	0.2	0.13	0.1	0.03
C3*-P1*	33.22	-64.43	1.08	0.12	35.85	-67.7	1.07	0.15	0.1	0.09	0.01
C3*-P2*	41.7	-81.4	1.2	0.13	47.05	-90.1	1.09	0.18	0.12	0.11	0.2
C3*-P3*	51.52	-101.04	1.36	0.15	60.38	-116.77	1.1	0.2	0.14	0.13	0.03
C5*-P1*	29.49	-56.97	1.04	0.11	28.44	-52.88	1.05	0.16	0.1	0.06	0.01
C5*-P2*	38.71	-75.43	1.18	0.13	40.57	-77.15	1.05	0.2	0.12	0.07	0.03
C5*-P3*	56.11	-110.23	1.44	0.16	64.91	-125.82	1.09	0.24	0.15	0.11	0.06
routes	Clayton Copula					Gumbel Copula					
	LLF	AIC	$\hat{\theta }$	K-*τ*	LTD	LLF	AIC	$\hat{a}$	K-*τ*	UTD	
C2*-P1*	27.54	-53.09	0.2	0.09	0.03	27.04	-52.08	1.09	0.09	0.12	
C2*-P2*	46.7	-91.8	0.27	0.12	0.08	43.25	-84.49	1.12	0.11	0.14	
C2*-P3*	39.36	-76.72	0.26	0.11	0.07	43.45	-84.91	1.12	0.11	0.15	
C3*-P1*	26.11	-50.22	0.2	0.09	0.03	31.49	-60.99	1.1	0.09	0.12	
C3*-P2*	34.93	-67.85	0.24	0.11	0.05	40.94	-79.89	1.12	0.1	0.14	
C3*-P3*	42.02	-82.04	0.27	0.12	0.08	53.98	-106.0	1.13	0.12	0.16	
C5*-P1*	24.02	-46.03	0.19	0.09	0.03	22.45	-42.91	1.09	0.08	0.11	
C5*-P2*	35.3	-68.6	0.24	0.11	0.05	29.9	-57.81	1.1	0.09	0.13	
C5*-P3*	51.98	-101.95	0.3	0.13	0.1	53.03	-104.1	1.14	0.13	0.17	

Note: K-*τ* stands for Kendall-*τ* and represents the correlation of freight rate returns pairs.

The largest static correlation is observed in the C5*-P3* pair, while the relatively small static correlations are found in C5*-P1*, C3*-P1*, and C2*-P1*. The linkage between the freight rate of the BCI market and the P1 route is notably low, which may be attributed to the varying impacts of different voyages on the correlation. The P1 route primarily serves as an Atlantic round-trip route, originating in the United States and terminating in Western Europe. In contrast, the C2, C3, and C5 routes do not originate from the United States, with both the C3 and C5 routes ultimately heading to China.

#### Dynamic Copula correlation analysis

To effectively illustrate the variation of the correlation coefficient over time, three types of time-varying Copula functions are selected to analyze the correlation between different freight rates. The parameters of these time-varying Copula functions are dynamically adjusted. This study employs the time-varying Normal (TVN) Copula, time-varying Rotated Gumbel (TVRG) Copula, and time-varying SJC (TVSJC) Copula for dynamic Copula modeling.

In Table 9, the time-varying SJC Copula is the optimal choice for all pairs except for C2*-P3*. The parameters of the time-varying SJC Copula are especially sensitive to tail data, allowing it to effectively capture asymmetric correlations in both the upper and lower tails. However, since the time-varying SJC Copula primarily focuses on characterizing tail correlation coefficients, we also employ the time-varying Normal Copula to better represent the overall symmetric correlation between route fares.

**Table 9:** AIC values of different dynamic Copula functions.

Routs	TVN Copula	TVRG Copula	TVSJC Copula
C2*-P1*	-66.554	-72.633	-78.615
C2*-P2*	-113.808	-110.498	-118.119
C2*-P3*	-113.676	-102.085	-111.461
C3*-P1*	-69.481	-68.219	-75.899
C3*-P2*	-99.725	-88.403	-103.959
C3*-P3*	-126.169	-112.204	-131.039
C5*-P1*	-58.234	-52.830	-60.904
C5*-P2*	-83.211	-77.662	-88.899
C5*-P3*	-126.079	-117.039	-132.727

From Table 10, it is evident that, except for C2*-P2*, C2*-P3*, and C5*-P2*, all other pairs exhibit positive correlations. Notably, C2*-P3* shows a few negative correlations during the early stages, which may be linked to the US interest rate hike in December 2015 and the collapse of the Chinese stock market around New Year’s Day in 2016. Both C2*-P2* and C5*-P2* display individual negative correlations in the later period, potentially due to the impacts of COVID-19. Examining the fluctuations in dynamic correlations, the standard deviations for C2*-P2* and C2*-P3* are relatively large, indicating frequent fluctuations. In contrast, the standard deviation of C5*-P1* is only 0.015, suggesting a relatively stable variation throughout the sample period.

**Table 10:** Descriptive statistics of time-varying Copula dynamic correlation coefficients.

routes	TVN Copula K-*τ*				TVSJC Copula upper tail K-*τ*				TVSJC Copula lower tail K-*τ*			
	mean	max	min	std	mean	max	min	std	mean	max	min	std
C2*-P1*	0.165	0.313	0.036	0.036	0.024	0.747	0.001	0.042	0.059	0.393	0.003	0.036
C2*-P2*	0.205	0.477	-0.041	0.069	0.035	0.306	0.002	0.019	0.100	0.375	0.008	0.030
C2*-P3*	0.215	0.415	-0.052	0.062	0.050	0.823	0.001	0.051	0.084	0.344	0.026	0.026
C3*-P1*	0.173	0.233	0.112	0.019	0.029	0.061	0.010	0.006	0.051	0.226	0.009	0.028
C3*-P2*	0.206	0.318	0.108	0.029	0.051	0.112	0.011	0.034	0.048	0.473	0.001	0.060
C3*-P3*	0.232	0.425	0.083	0.055	0.059	0.260	0.014	0.031	0.088	0.634	0.006	0.056
C5*-P1*	0.164	0.214	0.110	0.015	0.014	0.016	0.013	0.004	0.049	0.826	0.007	0.059
C5*-P2*	0.191	0.318	-0.021	0.041	0.020	0.893	0.001	0.053	0.086	0.355	0.021	0.026
C5*-P3*	0.243	0.375	0.121	0.031	0.056	0.285	0.003	0.033	0.110	0.301	0.050	0.022

**Fig 7:**
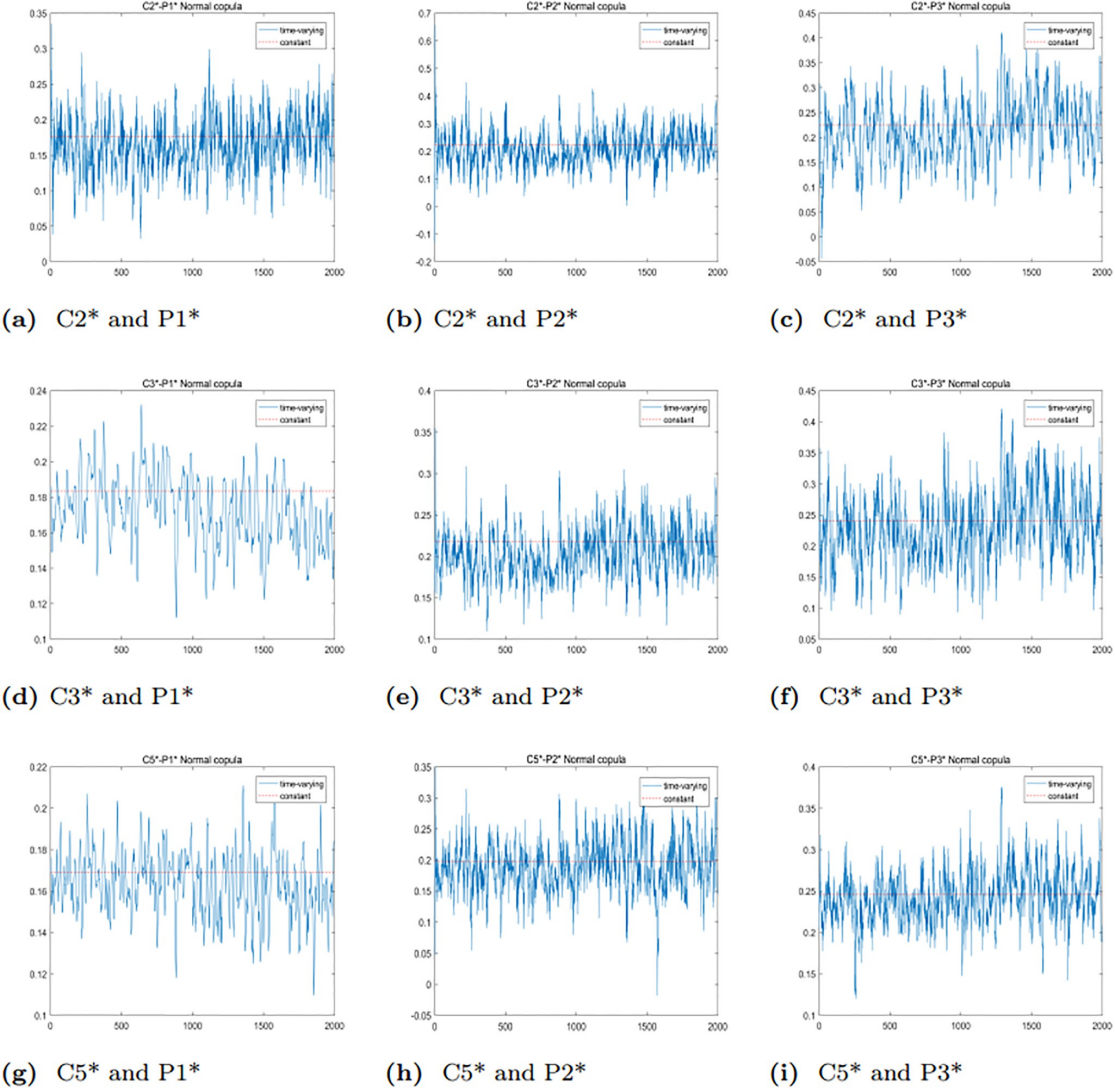
Trend plots of the TVN Copula dynamic correlation coefficients between the BCI and BPI markets.

The time series of dynamic correlation coefficients is illustrated in Fig 7. C5*-P3* demonstrates the largest mean dynamic correlation, while C5*-P1* shows the smallest mean dynamic correlation. This finding aligns with the conclusions drawn from the static optimal Copula analysis, indicating that the time-varying Normal Copula modeling performs well in capturing these relationships.

**Fig 8:**
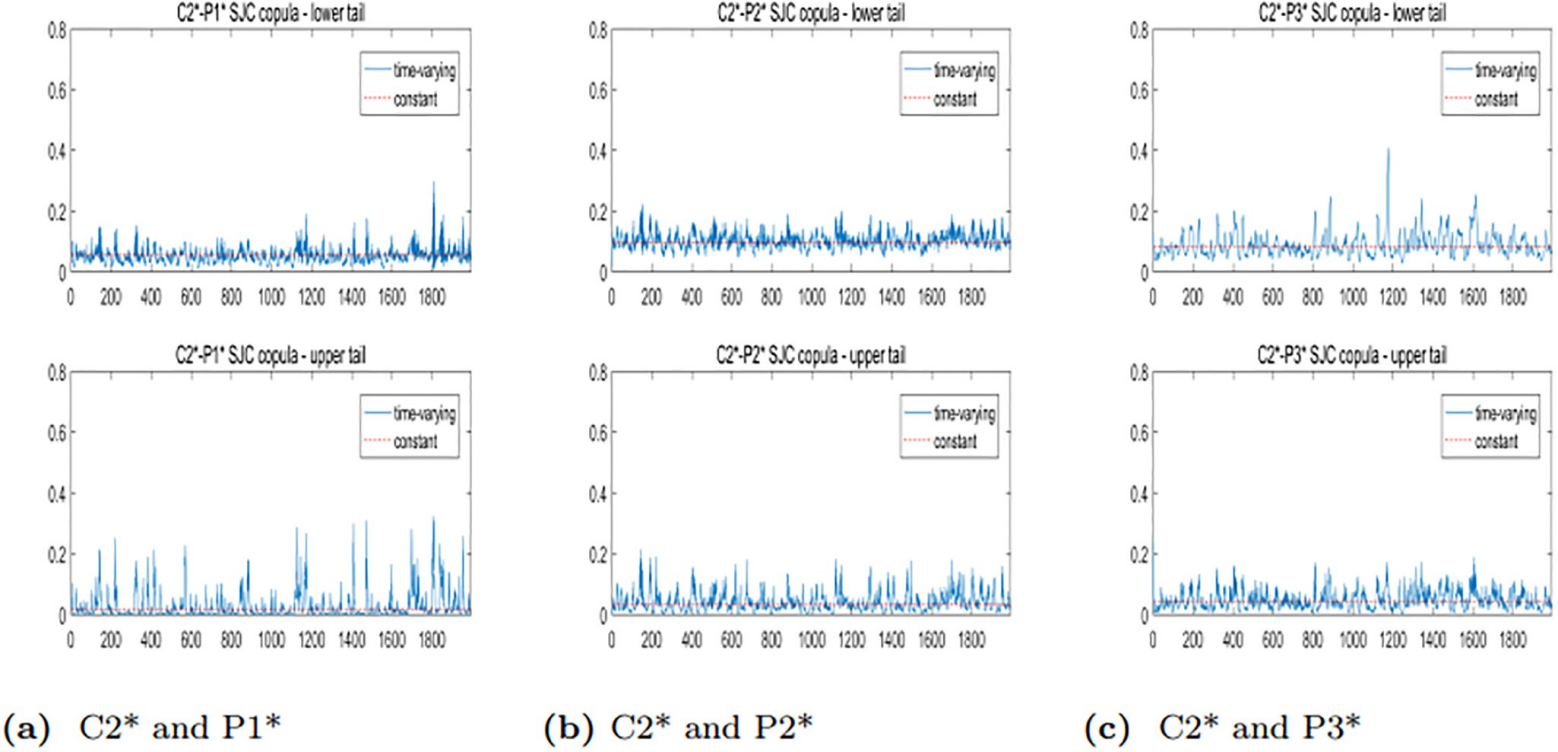
Trend plots of TVSJC Copula dynamic correlation coefficients between the C2* and BPI markets.

**Fig 9:**
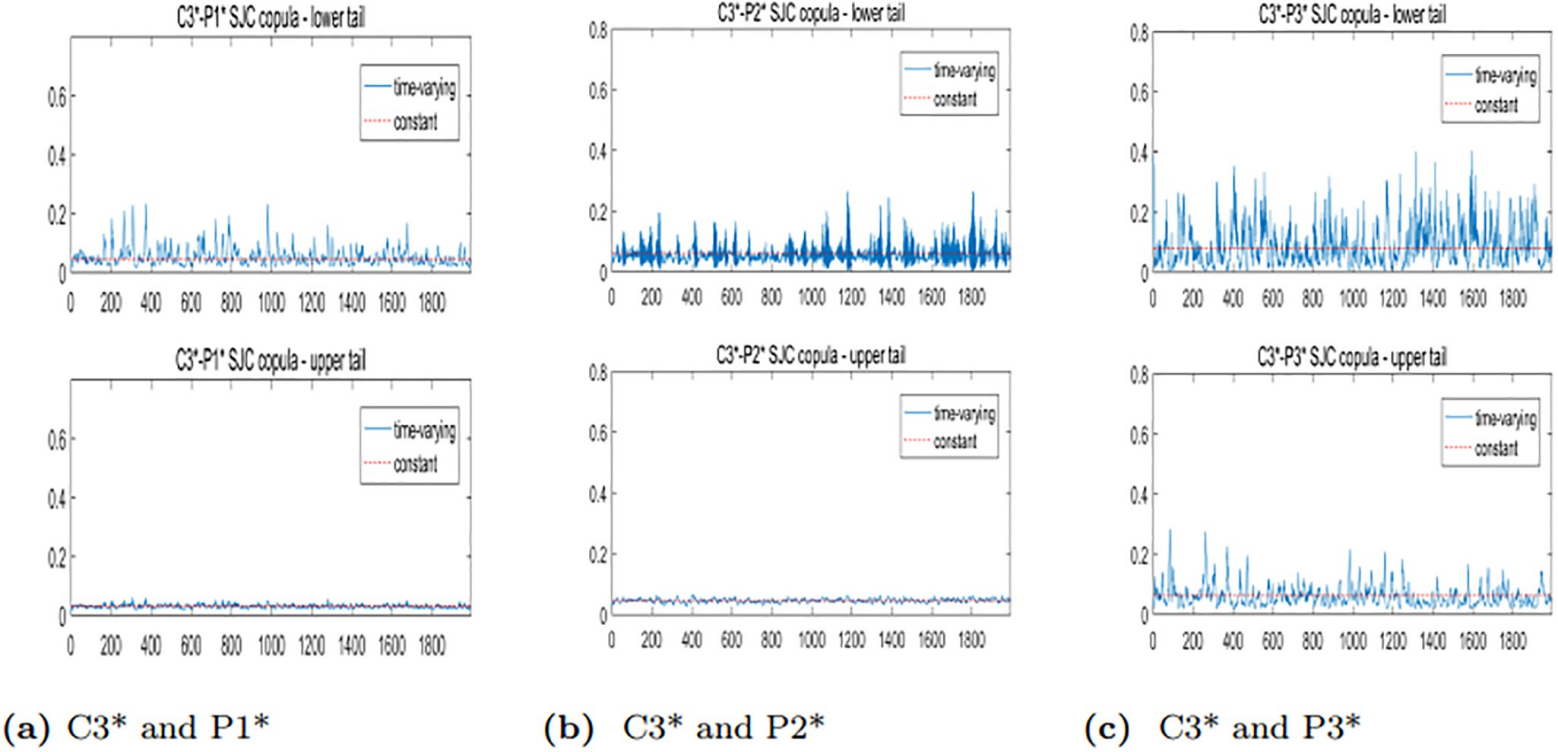
Trend plots of TVSJC Copula dynamic correlation coefficients between the C3* and BPI markets.

**Fig 10:**
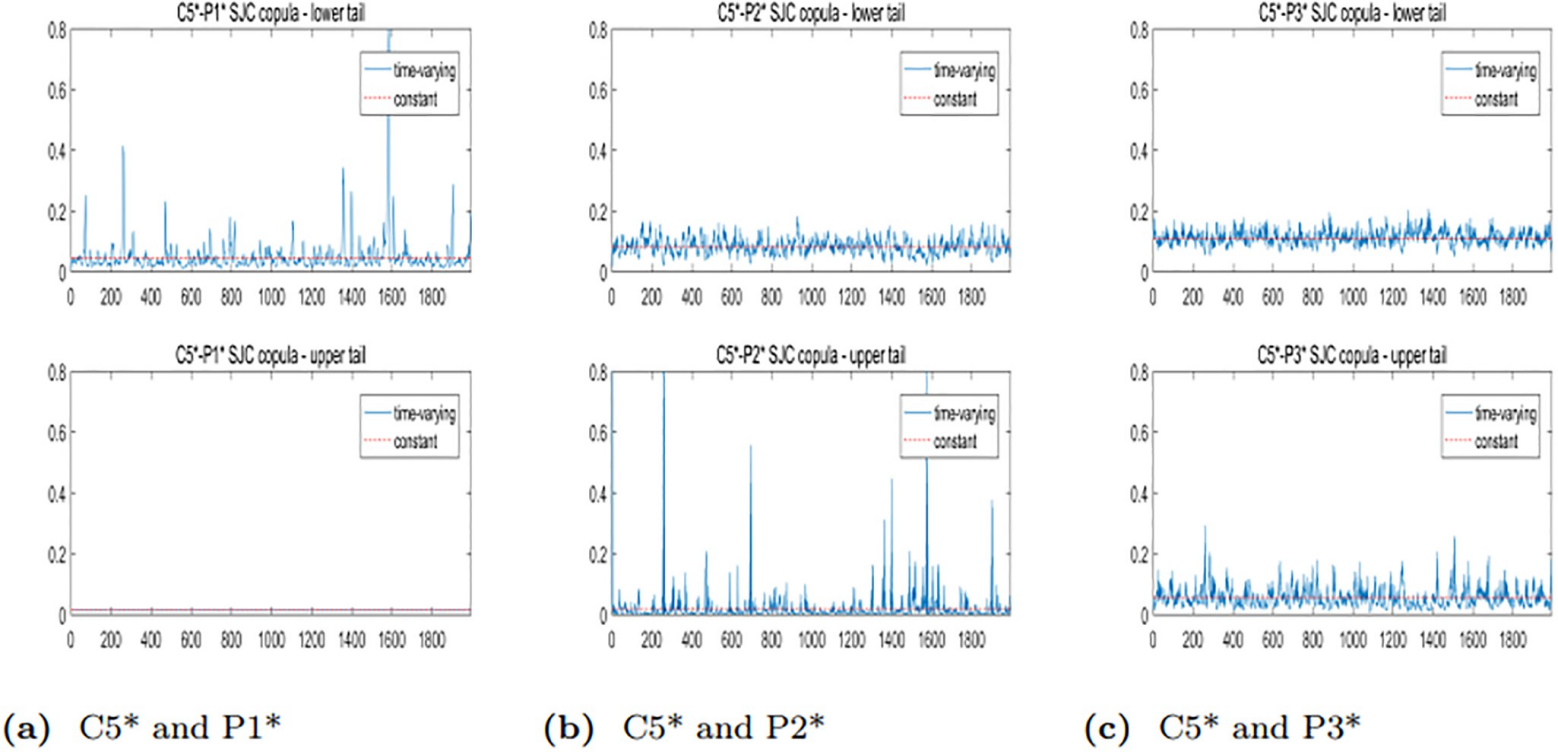
Trend plots of TVSJC Copula dynamic correlation coefficients between the C5* and BPI markets.

To further analyze the dynamic correlation between the upper and lower tails of freight rates for various routes in the BCI and BPI markets, we will employ the TVSJC Copula. As illustrated in Figs 8–10, the dynamic correlation coefficients exhibit asymmetric dependency structures. For all six pairs, the mean values of the lower tail correlation coefficients exceed those of the upper tail correlation coefficients. This suggests that the correlations between freight rates are stronger during market downturns or extreme crises. Additionally, there is a higher likelihood of simultaneous price drops while experiencing different price increases across freight routes in the BCI and BPI markets.

### Empirical analysis of risk spillover effect

#### Dynamic time-varying VaR results

**Fig 11:**
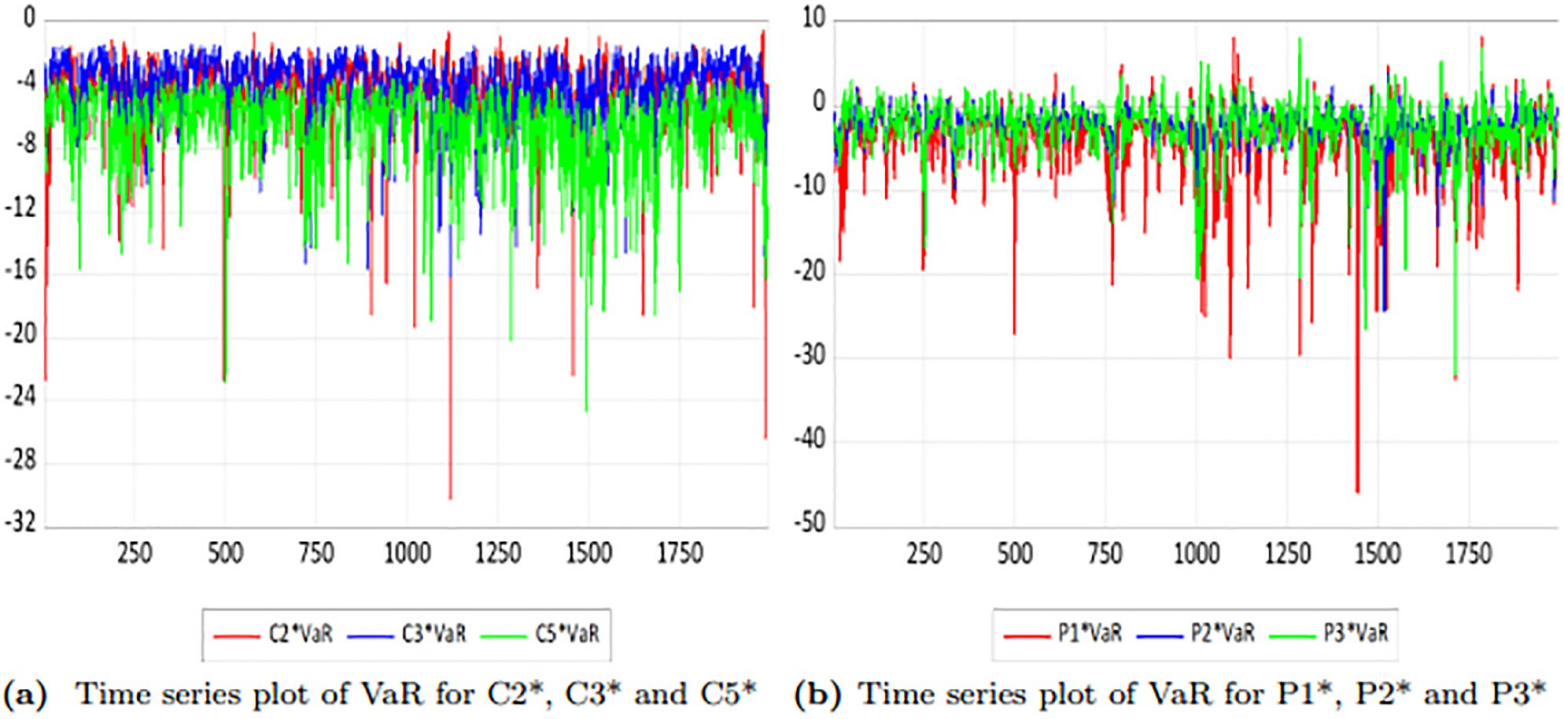
Trend plots of VaR for each returns pairs.

Before calculating CoVaR, it is essential to first compute VaR, as defined by the formula in (4). Fig 11 illustrates the dynamic time series of returns for each series. Table 11 presents the descriptive statistics of the time-varying VaR for each series.

**Table 11:** Descriptive statistics of time-varying VaR for each series (*α* = 0.05).

Statistics	C2*	C3*	C5*	P1*	P2*	P3*
mean	-4.238	-4.132	-6.647	-4.331	-2.061	-2.561
std	2.2030	2.087	2.425	4.2823	2.221	3.085

From Fig 11 and Table 11, it is evident that the VaR of each route’s freight rates exhibits time-varying characteristics with notable fluctuations. In the BCI market, the C3 route has a lower risk value compared to the other two routes, while the absolute VaR for the C5 route is the highest. This difference may be attributed to the C5 route, which runs from Western Australia to Qingdao, whereas the C3 route connects Tubarang (Brazil) to Qingdao. Australia consistently ranks first in global iron ore export volume, significantly surpassing Brazil and other regions. Consequently, trade fluctuations within the Australian domestic market and variations in iron ore exports may introduce potential risks to the freight rates of the C5 route.

In the BPI market, the P1 route exhibits the largest absolute value of average VaR, indicating that its fluctuations are the most significant. Conversely, the P2 route has the smallest absolute value of average VaR, suggesting its fluctuations are the least pronounced. This disparity may be attributed to the P2 route, which connects the Atlantic with the Far East (including China, Japan, and East Asia). In recent years, both China and Japan have implemented shipping trade policies that partially mitigate freight rate risks. Notably, the risk fluctuations for each route significantly increased in 2020 and 2022, likely due to the impacts of the global COVID-19 pandemic in 2020 and the Russia-Ukraine conflict in 2022. This underscores the influence of major global events on freight rates.

#### Dynamic time-varying CoVaR results

CoVaR measures the magnitude of mutual risk spillover effects between the BCI and BPI markets. Table 12 presents the descriptive statistics for the dynamic time-varying CoVaR of the BCI market as it relates to the BPI market, while Table 13 provides similar statistics for the BPI market in relation to the BCI market. From Tables 12 and 13, it is evident that the risks associated with the BCI and BPI routes have experienced different changes over time. Notably, the absolute value of the average VaR for the C2 route is 4.2383, as shown in Table 11, while the absolute value of the average CoVaR^P1|C2^ is 5.1869, the absolute value of the average CoVaR^P2|C2^ is 6.4045, and the absolute value of the average CoVaR^P3|C2^ is 5.2595, both of which exceed the VaR of the C2 route. This suggests that the VaR for the C2 route is likely underestimated, highlighting a limitation of relying solely on VaR to assess the risk of return series. Consequently, CoVaR is employed to measure the tail losses associated with VaR, providing a more comprehensive risk assessment.

**Table 12:** Descriptive statistics of dynamic time-varying CoVaR in BCI → BPI (*α* = 0.05).

	C2*→P1*	C2*→P2*	C2*→P3*	C3*→P1*	C3*→P2*	C3*→P3*	C5*→P1*	C5*→P2*	C5*→P3*
mean	-5.482	-3.194	-3.363	-5.548	-2.436	-3.450	-4.418	-2.671	-3.703
std	4.594	2.663	3.316	4.615	2.351	3.345	4.303	2.441	3.431

**Table 13:** Descriptive statistics of dynamic time-varying CoVaR in BPI → BCI (*α* = 0.05).

	P1*→C2*	P2*→C2*	P3*→C2*	P1*→C3*	P2*→C3*	P3*→C3*	P1*→C5*	P2*→C5*	P3*→C5*
mean	-5.186	-6.404	-5.259	-5.185	-4.692	-5.344	-7.165	-8.695	-9.512
std	2.417	2.740	2.435	2.378	2.237	2.424	2.534	2.873	3.062

**Fig 12:**
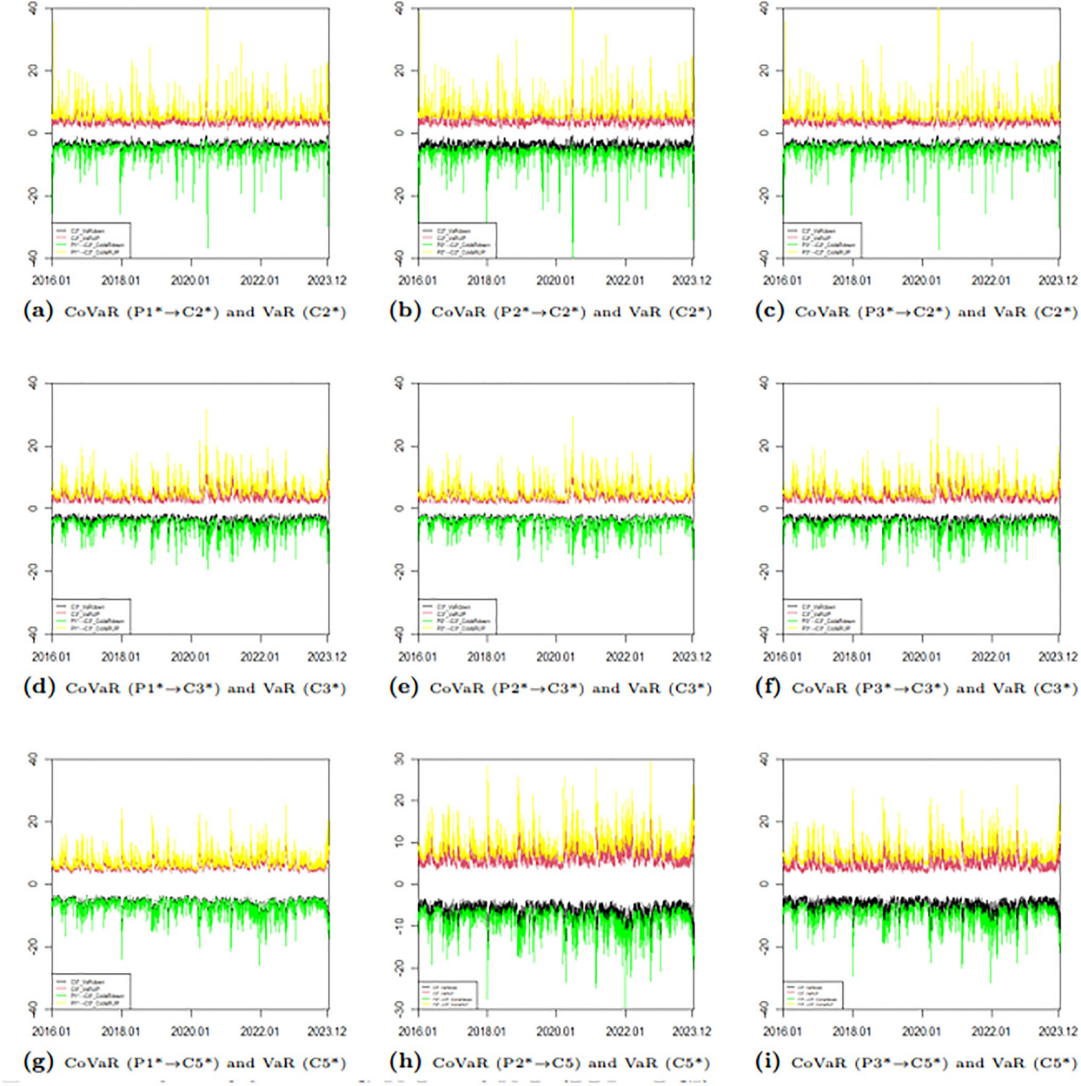
Time series plots of dynamic CoVaR and VaR (BPI→ BCI).

**Fig 13:**
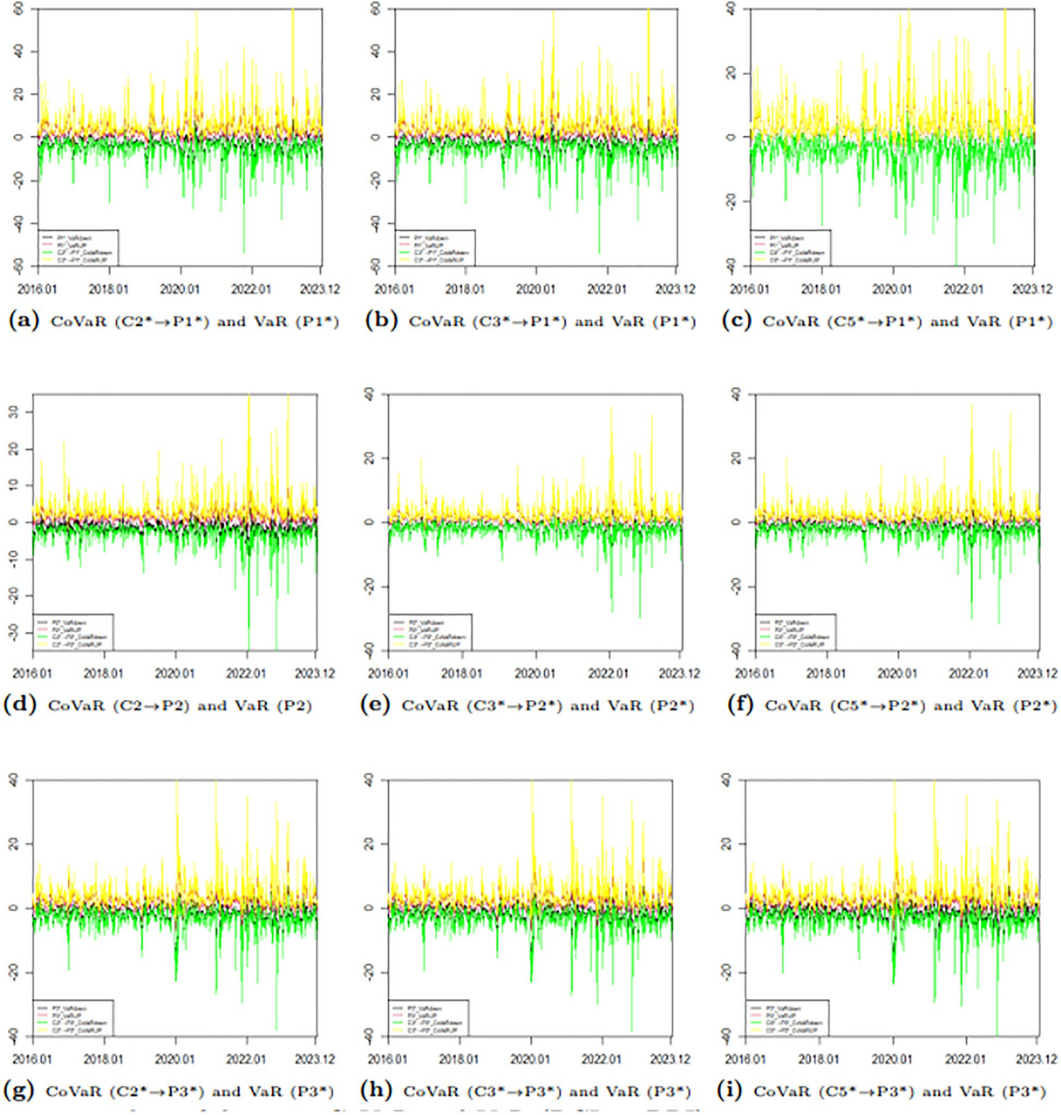
Time series plots of dynamic CoVaR and VaR (BCI → BPI).

To further illustrate the difference between VaR and CoVaR, we present the upward and downward risks of two markets. Fig 12 depicts the risk spillover effect of the BCI market on the BPI market, while Fig 13 shows the spillover effect of the BPI market on the BCI market. From Figs 12 and 13, it is clear that the VaR values for each route, as well as the CoVaR values for each pair, exhibit dynamic changes over time. Notably, the CoVaR from C5* to P1* largely overlaps with the VaR of P1*, indicating a one-way risk spillover from the freight rate of the P1 route to the freight rate of the C5 route. Conversely, the freight rate of the C5 route does not spill over risk to the freight index of the P1 route. Similarly, there is no spillover effect between the freight rates of the C3 and P2 routes. Additionally, the absolute values of the upward and downward CoVaRs are greater than their corresponding VaRs, which highlights the presence of ΔCoVaR and confirms the existence of bidirectional risk spillover.

#### Dynamic time-varying ΔCoVaR results

**Fig 14:**
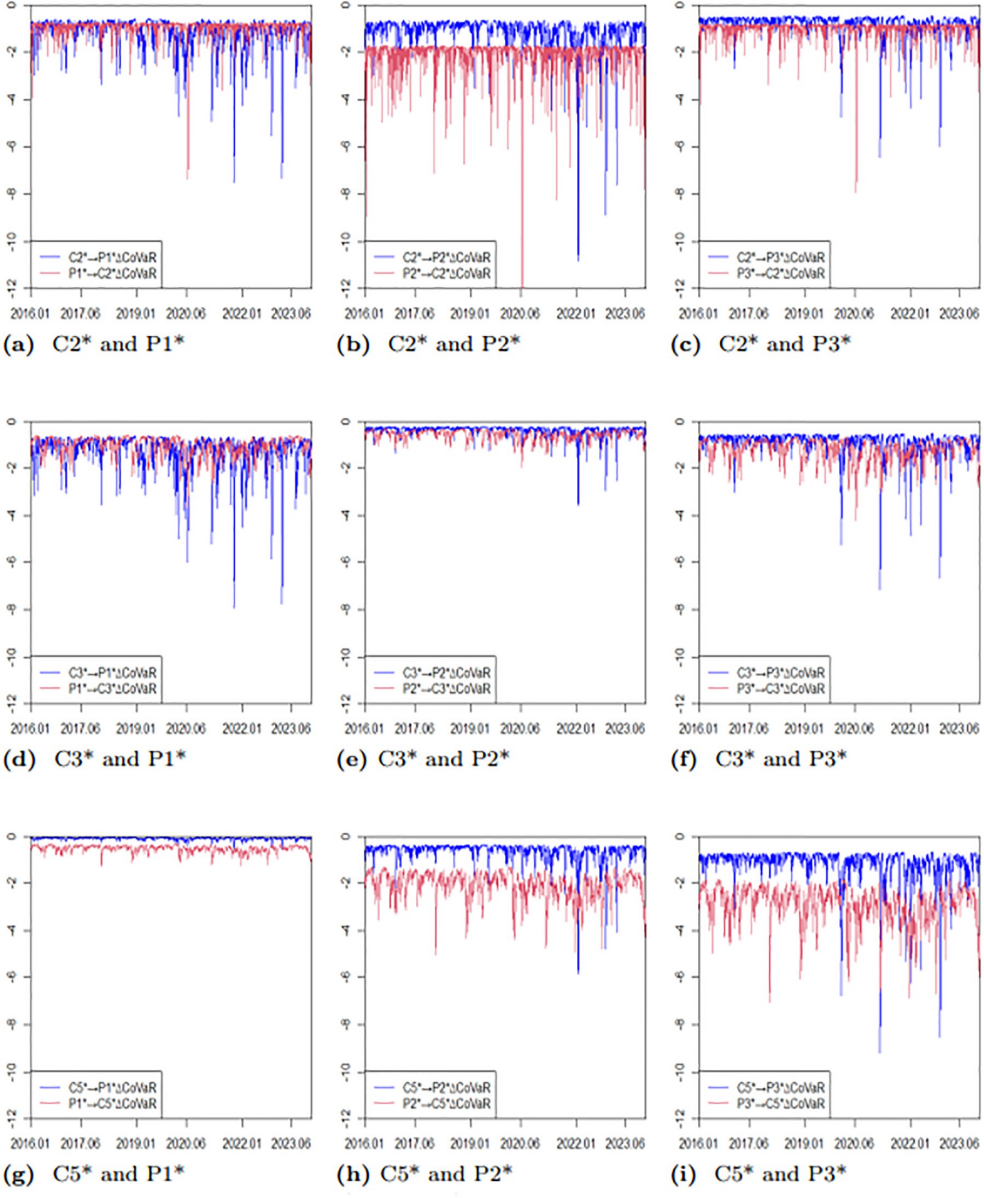
Time series plots of ΔCoVaR (*α* = 0.05).

This paper focuses on studying risk spillovers in extreme loss situations, specifically exploring the dynamic time-varying ΔCoVaR relationship between two markets under severe downward scenarios. The calculation formula for ΔCoVaR is provided in (6). Table 14 presents the descriptive statistical results for the dynamic time-varying ΔCoVaR from the BCI market to the BPI market, while Table 15 details the results from the BPI market to the BCI market. To better illustrate the intensity of risk spillover between different routes in the two markets, we include a time-varying plot of bidirectional risk spillover ΔCoVaR, as shown in Fig 14. From Tables 14 and 15, along with Fig 14, we can observe the following:

**Table 14:** Descriptive statistics of dynamic time-varying ΔCoVaR for freight rate returns (BCI→BPI).

	C2*→ P1*	C2*→P2*	C2*→P3*	C3*→P1*	C3*→P2*	C3*→P3*	C5*→ P1*	C5*→P2*	C5*→P3*
mean	-1.151	-1.133	-0.803	-1.218	-0.375	-0.890	-0.087	-0.610	-1.142
max	-0.589	-0.646	-0.464	-0.622	-0.214	-0.514	-0.044	-0.348	-0.660
min	-7.511	-10.827	-6.456	-7.944	-3.583	-7.156	-0.567	-5.824	-9.190
std	0.695	0.736	0.495	0.735	0.243	0.549	0.053	0.396	0.704

**Table 15:** Descriptive statistics of dynamic time-varying ΔCoVaR for freight rate returns (BPI→BCI).

	P1*→C2*	P2*→C2*	P3*→C2*	P1*→C3*	P2*→C3*	P3*→C3*	P1*→C5*	P2*→C5*	P3*→C5*
mean	-0.949	-2.166	-1.021	-1.054	-0.888	-1.213	-0.518	-2.048	-2.865
max	-0.751	-1.716	-0.809	-0.571	-0.481	-0.651	-0.327	-1.293	-1.808
min	-7.369	-16.827	-7.932	-3.658	-3.083	-4.211	-1.271	-5.024	-7.029
std	0.366	0.835	0.394	0.406	0.342	0.467	0.143	0.566	0.792

Firstly, the ΔCoVaR of freight rates for each route exhibits temporal fluctuations and some extreme values, highlighting the limitations of VaR in capturing risk spillovers. This confirms that VaR does not fully measure the complexities of risk interconnections.

Secondly, the risk spillover from P2 to C2 is significantly greater than that from C2 to P2. Similarly, the risk spillover from P2 to C5 exceeds that from C5 to P2, and the risk spillover from P3 to C5 is greater than that from C5 to P3. Notably, C5 has a relatively small risk spillover effect on P1, indicating a one-directional spillover between P1 and C5. This finding aligns with conclusions drawn in the previous section. With the exception of C5 and P1, all other pairs demonstrate two-directional and asymmetric risk spillover effects.

Thirdly, the absolute mean risk spillover from C3 to P1 is the largest. This can be attributed to the geographical proximity of the routes; both C3 and P1 are linked to the Atlantic, while C5 traverses the Pacific. Conversely, the mean risk spillover from C5 to P1 is the smallest, likely due to the greater distance between their respective starting points. Specifically, the distance of the C3 route is more than twice that of the C5 route. The mean risk spillover from P2 to C2 is the largest, while that from P1 to C5 is the smallest, further suggesting that route distances play a significant role in determining the magnitude of risk spillovers.

Fourthly, the ΔCoVaR values between -12 and 0 indicate that downward risk spillovers are consistently negative, and there is a positive correlation in the direction of risk between each freight rate pair. This suggests that freight rates in both the BCI and the BPI tend to move together, whether rising or falling.

Fifthly, although the time-varying ΔCoVaR plots differ among the routes, there is a clear trend: the absolute values of ΔCoVaR significantly increased from 2020 to 2022. This increase can be attributed to external shocks such as the COVID-19 pandemic and the Russia-Ukraine conflict, which have notably impacted risk spillovers between routes. This observation supports the argument that extreme events can substantially alter spillover effects.

#### Dynamic time-varying %CoVaR results

Although ΔCoVaR effectively measures risk spillover effects, it does not eliminate the influence of dimensionality. Therefore, we will proceed to calculate %CoVaR. This metric represents the relative strength of risk spillover effects and reflects the contribution of these effects between freight indices in two markets under extreme risk conditions. Table 16 presents the descriptive statistics for the downward dynamic time-varying %CoVaR from the BCI market to the BPI market, while Table 17 provides similar statistics for the downward dynamic time-varying %CoVaR from the BPI market to the BCI market. Additionally, to offer a clearer understanding of the intensity of risk spillover between each return pair, the rankings of the mean values for %CoVaR are displayed in Table 18.

**Table 16:** Descriptive statistics of dynamic time-varying %CoVaR for freight rate returns (BCI→BPI).

	C2*→P1*	C2*→P2*	C2*→P3*	C3*→P1*	C3*→P2*	C3*→P3*	C5*→P1*	C5*→P2*	C5*→P3*
mean	0.149	0.314	0.348	0.157	0.104	0.386	0.011	0.169	0.496
std	4.377	11.410	7.774	4.630	3.776	8.617	0.331	6.138	11.066

**Table 17:** Descriptive statistics of dynamic time-varying %CoVaR for freight rate returns (BPI → BCI).

	P1*→C2*	P2*→C2*	P3*→C2*	P1*→C3*	P2*→C3*	P3*→C3*	P1*→C5*	P2*→C5*	P3*→C5*
mean	0.262	0.597	0.282	0.285	0.151	0.328	0.082	0.322	0.451
std	0.161	0.369	0.174	0.126	0.067	0.145	0.019	0.074	0.104

**Table 18:** %CoVaR mean ranking result (*α* = 0.05).

Rank	Capesize → Panamax	Panamax → Capesize
1	C5*→P3*	P2*→C2*
2	C3*→P3*	P3*→C5*
3	C2*→P3*	P3*→C3*
4	C2*→P2*	P2*→C5*
5	C5*→P2*	P1*→C3*
6	C3*→P1*	P3*→C2*
7	C2*→P1*	P1*→C2*
8	C3*→P2*	P2*→C3*
9	C5*→P1*	P1*→C5*

From Tables 16–18, we can conclude that:

Firstly, all %CoVaR values are positive, indicating that extreme conditions affect both markets. Furthermore, there are significant differences in the intensity of risk spillovers among various return pairs.

Secondly, regarding the risk spillover from the BCI market to the BPI market, the freight rate of the C5 route contributes the most to the risk of the P3 route. This is likely due to the fact that both routes are connected to East Asia. Conversely, the C5 route has the least contribution to the risk spillover of the P1 route, primarily because there is less overlap between these two routes, which aligns with the findings of ΔCoVaR.

Thirdly, in the risk spillover from the BPI market to the BCI market, the freight rate of the P2 route has the greatest impact on the risk intensity of the C2 route. This relationship may be attributed to the distance of the routes; the P2 route is the longest among the three BPI internal routes, and longer distances tend to increase risk spillover intensity. On the other hand, the risk intensity of freight rates between the P1 route and the C5 route is the lowest, likely due to a lack of intersection between the two routes.

Finally, although the risk intensity from the P1 route to the C5 route is greater than that from the C5 route to the P1 route, the overall risk spillover intensity between these two routes is significantly lower than that of all other pairs.

## Section 6: Discussion

With the continuous development of the global economy, the scale and complexity of international trade are expanding. Freight fluctuations, as a crucial component of trade costs, significantly impact trade flow and economic growth. Therefore, analyzing the volatility of the BCI and BPI, along with their mutual influences, is essential for understanding the dynamics of the shipping economy and global trade. Moreover, the interaction between the shipping market and the financial market is growing. Freight rate fluctuations not only influence the share prices of shipping companies but also affect the performance of other financial assets. This study aims to uncover the risk spillover effects between the BCI and BPI, highlighting the broader implications for the shipping industry.

In recent literature, some have focus on the risk spillover relationships among shipping indices, futures, oil prices, and other assets [26–28], and some have investigated the risk spillover effects among various sub-segment maritime markets [12]. As far as we know, there is no literature to study the risk spillover among the freight rates of various routes in the shipping submarket. This paper deeply studies the mutual volatility and risk spillover effect of freight rates in the BCI and BPI markets. It explores how these indices influence each other and collectively reflect the health of the shipping market.

There are many innovative models that study risk spillovers in financial markets, such as GMM, GARCH-MIDAS-GAS-copula-CoVaR model [29], GARCH copula quantile regression model [30] and so on. However, GMM is applicable to the assumption that the data meets the homoscedasticity or normality, but the time series data adopted in this paper are heteroscedasticity and non-normality, which does not meet the assumptions of GMM. The GARCH-MIDAS-GAS-Copula can process data of different frequencies, while the time series data studied in this paper is of the same frequency. The GARCH copula quantile regression model focuses on risk spillover in a specific market state, while this paper focuses on systemic risk and its propagation in times of crisis. To sum up, the GARCH-Copula-CoVaR model is more suitable for the purpose of this paper.

It is suggested that the application scope of GARCH-Copula-CoVaR model can be broadened in future research to explore the differences in risk transmission in different market environments. Further research can consider more influencing factors (such as macroeconomic variables, geopolitical risks, etc.) to improve the accuracy of the model and the reliability of the research results. Using a dynamic panel data approach, we study how risk transmission changes over different time to better capture market dynamics. In addition, we can explore the combination of machine learning technology with traditional econometric models and statistical models to further improve the accuracy and applicability of risk prediction.

## Section 7: Conclusions and suggestions

The main purpose of this paper is to study the volatility, correlation and risk spillover effect between freight rates of main routes in BCI and BPI markets. This paper focuses on historical data to provide a clearer picture of the volatility and risk spillovers within the shipping market, rather than linking it to external data as most people have studied. Specific conclusions are drawn as follows: There is static and dynamic correlation between airline freight rates. In both static Copula and dynamic Copula, there are significant differences in correlation between different routes. The correlation between C5-P3A_03 was significantly higher than other combinations. There is significant risk spillover effect between route rates. There is a one-way risk spillover between P1A_03 and C5, while most routes show a two-way positive risk spillover. The distance and location of routes may be important factors that lead to the difference in risk spillover intensity between different routes. Based on these conclusions, this paper provides the following suggests.

(1) Suggestions to the government: 1) Strengthen oversight of the shipping market. The government should implement a comprehensive risk monitoring mechanism to identify and evaluate potential market risks promptly. Regular market reports should be issued, and transparent market data should be made available to enhance overall market visibility. 2) Establish a risk early warning system. An effective risk early warning system must be established to provide timely alerts during abnormal fluctuations in freight prices. This will enable relevant enterprises and investors to take proactive measures to mitigate risks. 3) Promote inter-industry collaboration. The government can encourage collaboration among shipping companies and between shipping lines and financial institutions. By fostering information sharing and resource integration, the overall risk management capacity of the industry can be significantly improved.
(2) Suggestions to shipping enterprises: 1) Implement advanced risk assessment models. Regular analysis of shipping market volatility should be conducted to identify risk spillovers between different routes. This proactive approach will help in better understanding and managing potential risks. 2) Provide professional training for practitioners. Focus on enhancing the risk management skills and response capabilities of industry professionals. This training will equip them to make swift and accurate decisions in a complex and fluctuating market environment. 3) Adopt a diversified portfolio strategy. Reduce reliance on specific routes by implementing a diversified investment strategy. This flexibility will allow for adjustments during market downturns, helping to avoid high-risk areas and optimize investment direction.
(3) Suggestions to investors: 1) During periods of significant freight rate fluctuations, investors closely monitor the dynamics of the Capesize and Panamax bulk carrier markets, adjusting their investment strategies in a timely manner to mitigate potential risks. 2) When the risk associated with a particular route increases, investors should consider reallocating funds to routes with lower risk spillovers, thereby reducing the overall risk exposure of their portfolio. 3) In a downturn of the dry bulk market, investors should flexibly adjust their portfolios to diversify risks. If the freight rate on a specific route drops significantly, they might consider investing in routes that exhibit relatively stable performance to achieve a better balance of returns.

## Supporting information

S1 Data (https://w.afbcs.cn/TM44q3). (XLSX)

## References

[1] Bai X, Lam JSL, Jakher A. Shipping sentiment and the dry bulk shipping freight market: New evidence from newspaper coverage. Transportation Research Part E: Logistics and Transportation Review. 2021;155:102490. doi: 10.1016/j.tre.2021.102490

[2] Bandyopadhyay A, Rajib P. The asymmetric relationship between Baltic Dry Index and commodity spot prices: evidence from nonparametric causality-in-quantiles test. Mineral Economics. 2023;36(2):217–237. doi: 10.1007/s13563-021-00287-y

[3] Ruan Q, Wang Y, Lu X, Qi J. Cross-correlations between Baltic Dry Index and crude oil prices. Physica A-Statistical Mechanics and its Applications. 2016;453:278–289. doi: 10.1016/j.physa.2016.02.018

[4] Karaoulanis I, Pelagidis T. Panamax markets behaviour: explaining volatility and expectations. Journal of Shipping and Trade. 2021;6:15. doi: 10.1186/s41072-021-00096-0

[5] Kumar A. Dynamics interrelationship in returns and volatilities among shipping freight markets. World Maritime University Dissertations. World Maritime University. 2016;1–67.

[6] Chang CC, Chou HC, Wu CC. Value-at-risk analysis of the asymmetric long-memory volatility process of dry bulk freight rates. Maritime Economics & Logistics. 2014;16(3):298–320. doi: 10.1057/mel.2014.13

[7] Ji Q, Bouri E, Roubaud D, Shahzad SJH. Risk spillover between energy and agricultural commodity markets: A dependence-switching CoVaR-copula model. Energy Economics. 2018;75:14–27. doi: 10.1016/j.eneco.2018.08.015

[8] Xu H, Tao BB, Shu Y, Wang Y. Long-term memory law and empirical research on dry bulks shipping market fluctuations. Ocean & Coastal Management. 2021;213:105838. doi: 10.1016/j.ocecoaman.2021.105838

[9] Yang J, Ge Y, Li K. Measuring volatility spillover effects in dry bulk shipping market. Transport Policy. 2022;125: 37–47. doi: 10.1016/j.tranpol.2022.01.018

[10] Bollerslev T. Generalized autoregressive conditional heteroskedasticity. Journal of econometrics. 1986;31(3):307–327. doi: 10.1016/0304-4076(86)90063-1

[11] Leonov Y, Nikolov V. A wavelet and neural network model for the prediction of dry bulk shipping indices. Maritime Economics & Logistics. 2012;14:319–333. doi: 10.1057/mel.2012.10

[12] Sun XL, Liu C, Wang J, Li J. Assessing the extreme risk spillovers of international commodities on maritime markets: a GARCH-Copula-CoVaR approach. International Review of Financial Analysis. 2020;68:101453. doi: 10.1016/j.irfa.2020.101453

[13] Fan YH, Xing YW, Yang HL. Prediction of Baltic Capesize Freight Index based on GARCH model. Applied Mechanics and Materials. 2014;488:1494–1497. doi: 10.4028/www.scientific.net/AMM.488-489.1494

[14] Riaz A, Xingong L, Jiao Z, Shahbaz M. Dynamic volatility spillover between oil and marine shipping industry. Energy Reports. 2023;9:3493–3507. doi: 10.1016/j.egyr.2023.02.025

[15] Lin AJ, Chang HY, Hsiao JL. Does the Baltic Dry Index drive volatility spillovers in the commodities, currency, or stock markets? Transportation Research Part E: Logistics and Transportation Review. 2019;127:265–283. doi: 10.1016/j.tre.2019.05.013

[16] Schindler D, Behr HD, Jung C. On the spatiotemporal variability and potential of complementarity of wind and solar resources. Energy Conversion and Management. 2020; 218:113016. doi: 10.1016/j.enconman.2020.113016

[17] Chen FE, Tian K, Miao YQ, Li T, Ding X. Multifractal characteristics in maritime economics volatility. International Journal of Transport Economics. 2017,44(3):365–380. doi: 10.19272/201706703001

[18] Adrian T, Brunnermeier MK. CoVaR. American Economic Review. 2016;106(7):1705–1741. doi: 10.1257/aer.20120555

[19] Giamouzi M, Nomikos NK. Identifying shipowners’ risk attitudes over gains and losses: Evidence from the dry bulk freight market. Transportation Research Part E: Logistics and Transportation Review. 2021;145:102129. doi: 10.1016/j.tre.2020.102129

[20] Ji Q, Liu BY, Fan Y. Risk dependence of CoVaR and structural change between oil prices and exchange rates: A time-varying copula model. Energy Economics. 2019;77:80–92. doi: 10.1016/j.eneco.2018.07.012

[21] Ji Q, Liu BY, Nehler H, Uddin GS. Uncertainties and extreme risk spillover in the energy markets: A time-varying copula-based CoVaR approach. Energy Economics. 2018;76:115–126. doi: 10.1016/j.eneco.2018.10.010

[22] Bildirici M. The chaotic behavior among the oil prices, expectation of investors and stock returns: TAR-TR-GARCH Copula and TAR-TR-TGARCH Copula. Petroleum Science. 2019;16(01):217–228. doi: 10.1007/s12182-018-0281-7

[23] Sharma U, Karmakar M. Are gold, USD, and Bitcoin hedge or safe haven against stock? The implication for risk management. Review of Financial Economics. 2023;41(1):43–64. doi: 10.1002/rfe.1160

[24] Wu RR, Qin ZF. Assessing the extreme risk spillovers to carbon markets from energy markets: evidence from China. Environmental Science and Pollution Research. 2022;30(13):37894–37911. doi: 10.1007/s11356-022-24610-4 36576632

[25] Meng L., Bin W. Risk spillovers and extreme risk between e-commerce and logistics markets in China. AIMS Mathematics. 2024;9(10): 29076–29106. doi: 10.3934/math.20241411

[26] Beenstock M, Vergottis A. An econometric model of the world market for dry cargo freight and shipping. Applied Economics. 1989;21(3):339–356. doi: 10.1080/758522551

[27] Norman VD, Wergelnd T. Nortank: a simulation model of the freight market for large tankers. Bergen: Norwegian School of Economics and Business Administration. 1981.

[28] Strandenes SP. Norship: a simulation model for bulk shipping markets. Bergen: Norwegian School of Economics and Business Administration. 1986.

[29] Yao C, Li M. GARCH-MIDAS-GAS-copula model for CoVaR and risk spillover in stock markets. The North American Journal of Economics and Finance. 2023; 66: 101910. doi: 10.1016/j.najef.2023.101910

[30] Tian M, Guo F, Niu R. Risk spillover analysis of China’s financial sectors based on a new GARCH copula quantile regression model. The North American Journal of Economics and Finance. 2022;63, 101817. doi: 10.1016/j.najef.2022.101817

